# Organoid Modeling of Mouse Anterior Tongue Epithelium Reveals Regional and Cellular Identities

**DOI:** 10.1002/advs.202506738

**Published:** 2025-09-29

**Authors:** Seok‐Young Kim, Laurens H. G. Verweij, Lin Lin, Johan H. van Es, Jay Slack, Chris Winkel, Tito Candelli, Philip Lijnzaad, Thanasis Margaritis, Gerben E. Breimer, Karin Sanders, Marc van de Wetering, Hans Clevers

**Affiliations:** ^1^ Princess Máxima Center for Pediatric Oncology Utrecht 3584 CS The Netherlands; ^2^ Hubrecht Institute Royal Netherlands Academy of Arts and Sciences (KNAW) Utrecht 3508 AD The Netherlands; ^3^ Department of Science + Technology Givaudan Flavors Corp Cincinnati OH 45216 USA; ^4^ Givaudan Nederland Flavors Naarden 1411 GP The Netherlands; ^5^ Department of Pathology University Medical Center Utrecht Utrecht 3508 GA The Netherlands; ^6^ Roche Pharmaceutical Research and Early Development (pRED) of F. Hoffmann‐La Roche Ltd CH‐4070 Basel Switzerland

**Keywords:** dorsal tongue, epithelial cell differentiation, organoids, single‐cell RNA sequencing

## Abstract

The tongue is essential for swallowing, taste perception, and mechanosensation. The anterior and posterior parts of the tongue have region‐specific developmental origins and are maintained by adult epithelial stem/progenitor cells. In vitro models that can be used to investigate anterior tongue biology have been lacking. Here, a protocol is developed to generate a long‐term expanding organoid model from the adult mouse dorsal anterior tongue. Anterior tongue organoids consist of *Lgr6*+ cells, *Sox2*+ stem/progenitor cells, and *Hoxc13*+ filiform papillae progenitor cells. Furthermore, anterior tongue organoids share region‐specific transcriptomic profiles, gene regulatory networks, and signaling pathways with anterior tongue tissue. Anterior tongue organoids can be differentiated into various epithelial cell types, including Merkel‐like cells, keratinocytes, and taste bud cells. Gene regulatory network analysis reveals transcriptional programs associated with *Krt8*+ cell and *Krt23+/Sbsn+* keratinocyte differentiation in the organoids. Together, this study provides an in vitro model of mouse dorsal tongue epithelium.

## Graphical abstract

The tongue's anterior epithelium is maintained by region‐specific stem/progenitor cells, yet in vitro models are limited. Long‐term expanding organoids are developed from adult mouse anterior tongue that retain region‐specific identity, cellular diversity, and gene regulatory features. These organoids differentiate into keratinocytes, Merkel‐like cells, and taste bud cells, providing a robust model to study anterior tongue biology.

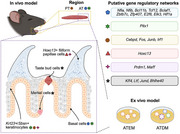

## 1 Introduction

The tongue is a multi‐functional sensorimotor organ that is essential for swallowing, taste, and texture perception.^[^ ^1^ ^]^ The dorsal surface of the adult mouse tongue harbors various types of epithelial cells, including basal cells, leucine‐rich repeat‐containing G protein‐coupled receptor 5‐positive (*Lgr5*+) and Lgr6+ stem cells, taste bud cells, minor salivary gland cells, keratinocytes, and Merkel cells.^[^ ^2^, ^3^, ^4^, ^5^, ^6^ ^]^ Interestingly, the presence of some of these epithelial cells is region‐specific. For example, Lgr5+ stem cells are exclusively found in the posterior part of the tongue, whereas Lgr6+ stem cells occur in both the posterior and the anterior part of the tongue.^[^ ^4^, ^7^ ^]^ Circumvallate papillae (CvP) and foliate papillae (FoP), distinct structures that harbor taste buds, are located in the posterior tongue (PT) and have an endodermal origin.^[^ ^8^ ^]^ In contrast, fungiform papillae (FP), another type of papillae that harbor taste buds, are located in the anterior tongue (AT) and have an ectodermal origin. Furthermore, minor salivary glands and filiform papillae, a type of papillae without taste buds, have both endodermal and ectodermal origins.^[^ ^8^ ^]^ Previous studies have highlighted the cellular and anatomical complexity of the tongue. Yet, it remains unclear whether AT and PT epithelia have distinct transcriptomic profiles and how regional or cellular identity is determined.

Organoids are 3D adult stem cell‐derived cell cultures that phenocopy the tissues from which they were generated.^[^ ^7^ ^]^ Organoids are grown in a defined medium containing an optimized growth factor cocktail and are surrounded by a basement membrane‐like hydrogel, called Matrigel/base membrane extract (BME).^[^ ^7^, ^9^ ^]^ Previous studies have demonstrated that organoids can be generated from mouse PT tissues (termed “PT organoids in expansion medium” or “PTEM”) using organoid medium containing R‐spondin 1, Noggin, and EGF.^[^ ^4^, ^10^, ^11^ ^]^ These organoids harbor taste bud cells, salivary gland cells, and keratinocytes.^[^ ^4^, ^10^, ^11^, ^12^ ^]^ Additionally, keratinocyte organoids lacking the taste bud compartment have been generated.^[^ ^13^ ^]^ However, no AT organoids capable of long‐term passaging have been reported.

In this study, we developed a method to generate organoids from mouse AT epithelium. By modulating organoid medium and performing single‐cell RNA sequencing of organoids and tissues, we explore cellular heterogeneity and the underlying transcriptional programs in the anterior tongue epithelium.

## 2 Results

### 2.1 Establishment of Mouse AT Organoids

**Figure 1:**
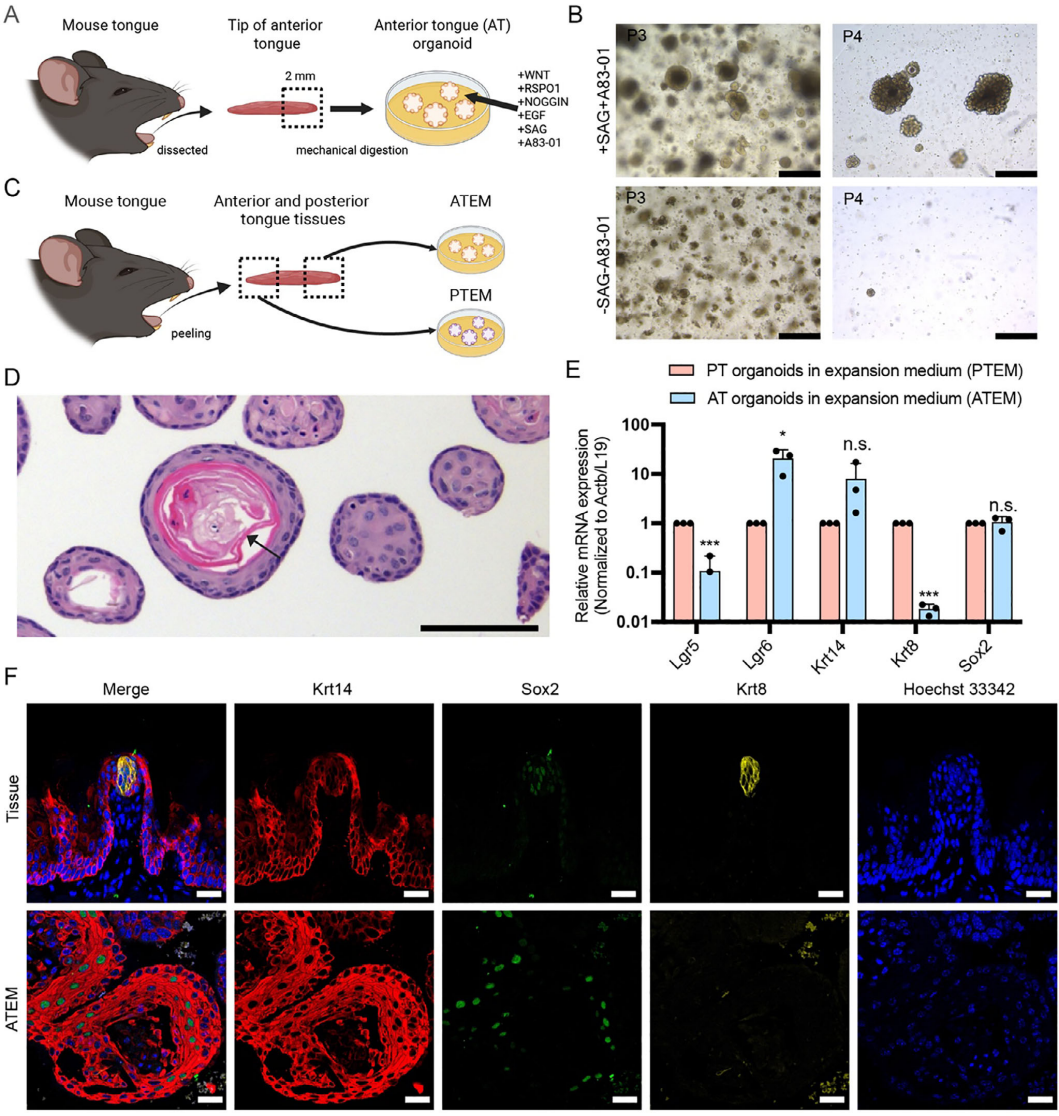
Generation and characterization of mouse AT organoids. A) A workflow to generate AT organoids from mouse tissue. A ≈2 mm tip of the anterior tongue tissue was minced and embedded in BME to generate the AT organoids. The AT organoids cultured in the described expansion medium were termed “ATEM”. B) Representative brightfield images of organoids in ATEM with (top row) or without (bottom row) SAG and A83‐01. Scale bar, 500 µm. P = passage number. C) A workflow to generate ATEM and posterior tongue organoids in expansion medium (termed “PTEM”) from mouse anterior and posterior tongue tissues, respectively. Circumvallate papillae and foliate papillae tissues were collected using tweezers, minced, and embedded in BME to generate PTEM. D) A representative hematoxylin and eosin image of ATEM. The black arrow indicates the previously described stratum corneum. Scale bar, 500 µm. E) Quantitative PCR comparing cell type markers in ATEM and PTEM (two‐tailed *t*‐test: *p < 0.05; ***p < 0.0001; n.s., non‐significant). Results from technical triplicates of biological triplicates (individual dots) are shown. F) Representative fluorescent immunohistochemistry images of mouse AT tissue and ATEM. Tissues and organoids were stained with the described markers. Results from biological duplicates are shown. Hoechst 33 342 (blue) marks the nuclei. Scale bar, 50 µm. AT = anterior tongue; PT = posterior tongue.

We aimed to develop a protocol to establish organoids from mouse AT tissue (**Figure** **1**A).^[^ ^4^, ^10^ ^]^ In brief, the first ≈2 mm from the tip of the mouse tongue tissue was collected to avoid contamination with PT tissue. Then, tissues were minced, embedded in BME, and cultured in organoid expansion medium including Wnt, R‐spondin 1, Noggin, EGF, A83‐01, TGFBR inhibitor, and Smoothened agonist (SAG; Sonic hedgehog pathway activator).^[^ ^14^ ^]^ As optimization of a previously published medium, A83‐01 and SAG were included because A83‐01 supports long‐term growth of various types of organoids,^[^ ^15^ ^]^ and Shh activation is important for papillae homeostasis.^[^ ^16^, ^17^, ^18^, ^19^, ^20^ ^]^ These organoids, termed “AT organoids in expansion medium” (ATEM) could be stably cultured over multiple passages (>P15). Notably, SAG and A83‐01 were essential for long‐term passaging (Figure 1B).

Organoids are known to recapitulate the cellular and molecular characteristics of the tissues from which they are derived.^[^ ^7^ ^]^ Lgr6 is a marker for adult taste stem cells in AT tissue, whereas Lgr5 and/or Lgr6 are markers for adult taste stem cells in PT tissue.^[^ ^4^ ^]^ Thus, we investigated whether ATEM represents the different cell populations and structures that were present in the previously reported PTEM (*4*) (Figure 1C). In brief, mouse CvP and FoP tissues were collected using tweezers and finely minced before being embedded in BME and cultured in R‐spondin 1, Noggin, and EGF.^[^ ^4^ ^]^ In histological analysis, ATEM consisted of organoids that harbored stratum corneum structures (sometimes with a lumen), the frequency of which varied among lines (Figure 1D, Figure S1A,B, Supporting Information).^[^ ^13^ ^]^ Compared to ATEM, PTEM harbored (pseudo)gland‐like structures (Figure S1C, Supporting Information). ATEM had significantly higher mRNA expression of *Lgr6* and lower expression of Lgr5 than PTEM (Figure 1E).^[^ ^4^ ^]^ mRNA expression levels of *Krt14*, a basal cell marker, and *Sox2*, a taste competent stem/progenitor cell marker,^[^ ^21^ ^]^ were comparable between ATEM and PTEM. *Krt8* (a marker for taste bud cells, minor salivary gland cells, and Merkel cells) was significantly less expressed in ATEM compared to PTEM. Immunostaining confirmed strong Krt14 and Sox2 expression and virtually absent Krt8 expression in ATEM (Figure 1F). Together, these results implied that AT organoids are capable of long‐term passaging and harbor region‐specific stem/progenitor cells.

### 2.2 AT Organoids Retain AT Tissue‐Specific Gene Signature

**Figure 2:**
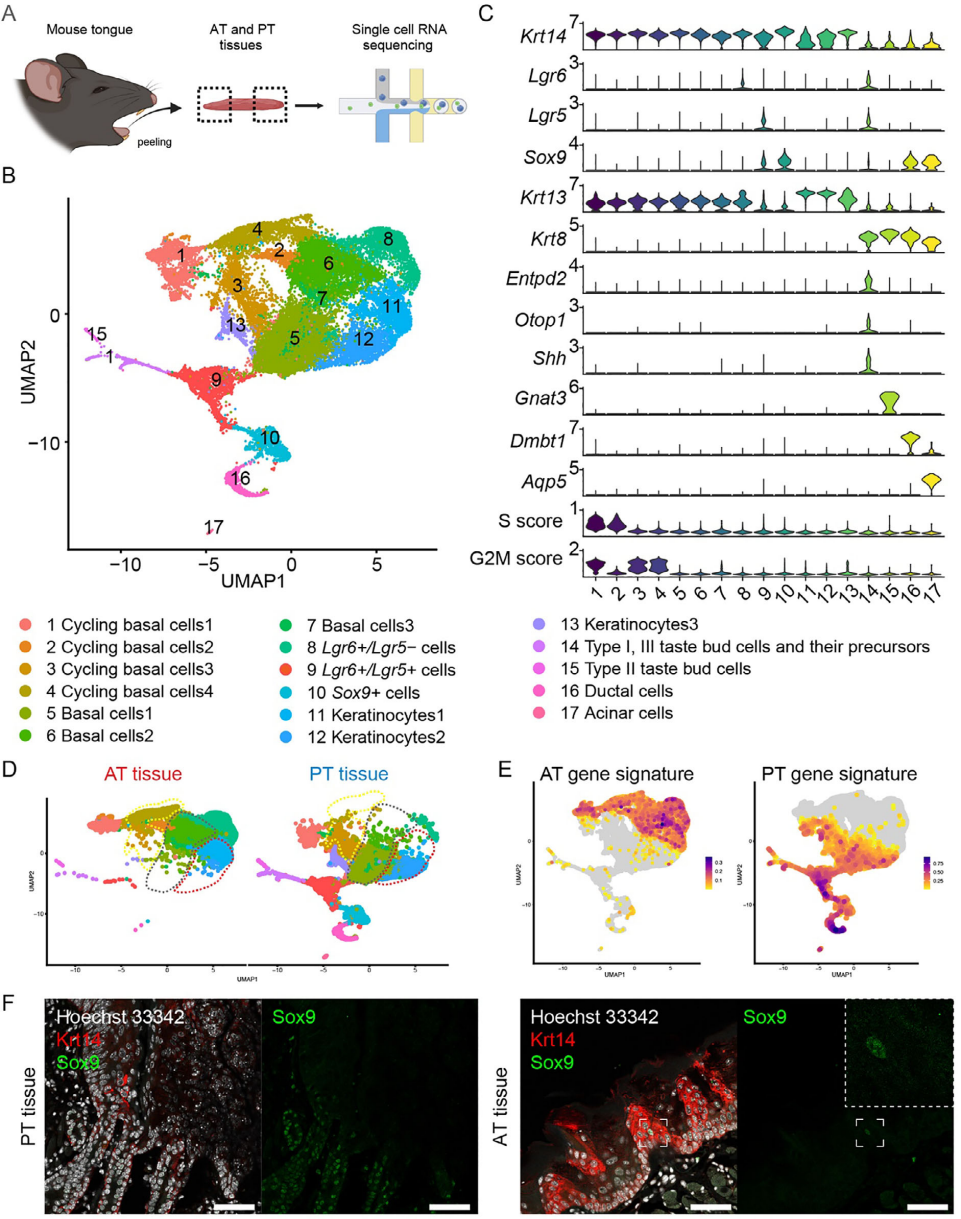
Mouse anterior and posterior dorsal tongue tissues display region‐specific gene signatures at a single‐cell level. A) A workflow to process mouse anterior tongue (AT) and posterior tongue (PT) tissues for single‐cell RNA sequencing (scRNA‐seq). B) UMAP visualization of 17 epithelial cell clusters in the tissue scRNA‐seq data. C) A violin plot showing expression of known markers and cell cycle scores for each cluster. In B,C) clusters are color‐coded. D) UMAP split by tissue origins of each sample. Cycling basal cells (yellow dotted line), basal cells (grey dotted line), and keratinocytes (red dotted line) are segregated by sample origin. E) UMAP showing AT and PT gene signature patterns. F) Representative fluorescent immunohistochemistry images showing expression of Sox9 (green) and Krt14 (red) in PT and AT tissues. Sox9+ cell in the AT tissue is zoomed in. Hoechst 33 342 (white) marks the nuclei. Results from biological duplicates are shown. Scale bar, 50 µm. AT = anterior tongue; PT = posterior tongue.

We further investigated the cellular and molecular heterogeneity of the organoids (ATEM and PTEM) and tissues (AT and PT) using 10X single‐cell mRNA sequencing (scRNA‐seq). First, we generated a reference dataset by performing scRNA‐seq on mouse tissues (AT and PT) (**Figure** **2**A). After quality control, we obtained 29 608 cells, including epithelial cells, immune cells, and stromal cells (AT tissue: 13 614 cells, PT tissue: 15 994 cells) (Figures S2A–D and S3, Supporting Information). Then, epithelial cells (n = 28 125) were used for downstream scRNA‐seq analysis (Figure 2B). We identified cycling basal cells and basal cells (*Krt14+*), *Lgr6+/Lgr5‐* cells, *Lgr6+/Lgr5+* cells, *Sox9+* cells, keratinocytes (*Krt13+*), taste bud cells (*Krt*8+, *Entpd2+, Otop1+, Gnat3+*) and minor salivary gland cells (ductal cells (*Dmbt1+*) and acinar cells (*Aqp5+*)) based on expression of known marker genes and cell cycle scores (Figure 2C). While Lgr5 protein expression was not detected in the AT tissue,^[^ ^3^, ^22^ ^]^ *Lgr5* mRNA expression was clearly observed (Figure 2D; Figures S2D and S3, Supporting Information), in agreement with our observation in ATEM (Figure 1E) and with Ren et al.^[^ ^4^ ^]^

We noted a high degree of region restriction in some Uniform Manifold Approximation and Projection for Dimension Reduction (UMAP) clusters. Basal cells and keratinocytes, while maintaining their very well‐known cell type markers (*Krt14* and *Krt13*, respectively)(Figure 2A,C),^[^ ^2^, ^23^ ^]^ clustered separately based on their tissue origins (Figure 2D). Furthermore, *Lgr6+/Lgr5+* cells and *Lgr6+/Lgr5‐* cells were restricted to PT and AT tissues, respectively (Figure 2D). We thus investigated which genes are differentially expressed between these AT and PT enriched clusters (cluster 2, 4, 6, 7, 8, and 11 vs cluster 3, 5, 9, 12, 13) (Figure 2A,D). We identified 85 and 45 differentially expressed genes (DEG; log fold change of ≥ 1.5 to 3‐fold difference in the percentage of cells expressing the gene) for each group, which we termed “AT gene signature” and “PT gene signature”, respectively (Figure 2E; Figure S4A,B, Supporting Information). For example, *Pitx2*, present in the AT gene signature, was prevalent in basal cells and keratinocytes of the AT tissue (Figure S4A, Supporting Information). Pitx2 was highly expressed in the AT epithelium, whereas it was not expressed in the PT epithelium (Figure S4C, Supporting Information). *Sox9*, present in the PT gene signature, was highly expressed in *Lgr6+/Lgr5+* cells, *Sox9+ basal cells, and minor salivary gland cells* (Figure S4B, Supporting Information).^[^ ^10^ ^]^ Accordingly, Sox9 was highly expressed in the base of the PT trench area where these cells reside, while it was sporadically and weakly expressed in the anterior tongue epithelium (Figure 2F). We asked whether these region‐specific gene signatures were imprinted in different germ cell layers during embryogenesis.^[^ ^24^ ^]^ To this end, we examined gene expression patterns in previously described scRNA‐seq data of mouse embryos from 6.5 to 8.5 days post‐fertilization, capturing key phases of early organogenesis.^[^ ^25^ ^]^ Our analysis showed that the AT and PT signatures were not associated with a specific germ layer (Figure S5A,B, Supporting Information), suggesting that the region identity may be determined later during embryogenesis. Together, these results demonstrated that AT and PT epithelial cells display distinct gene expression features.

**Figure 3:**
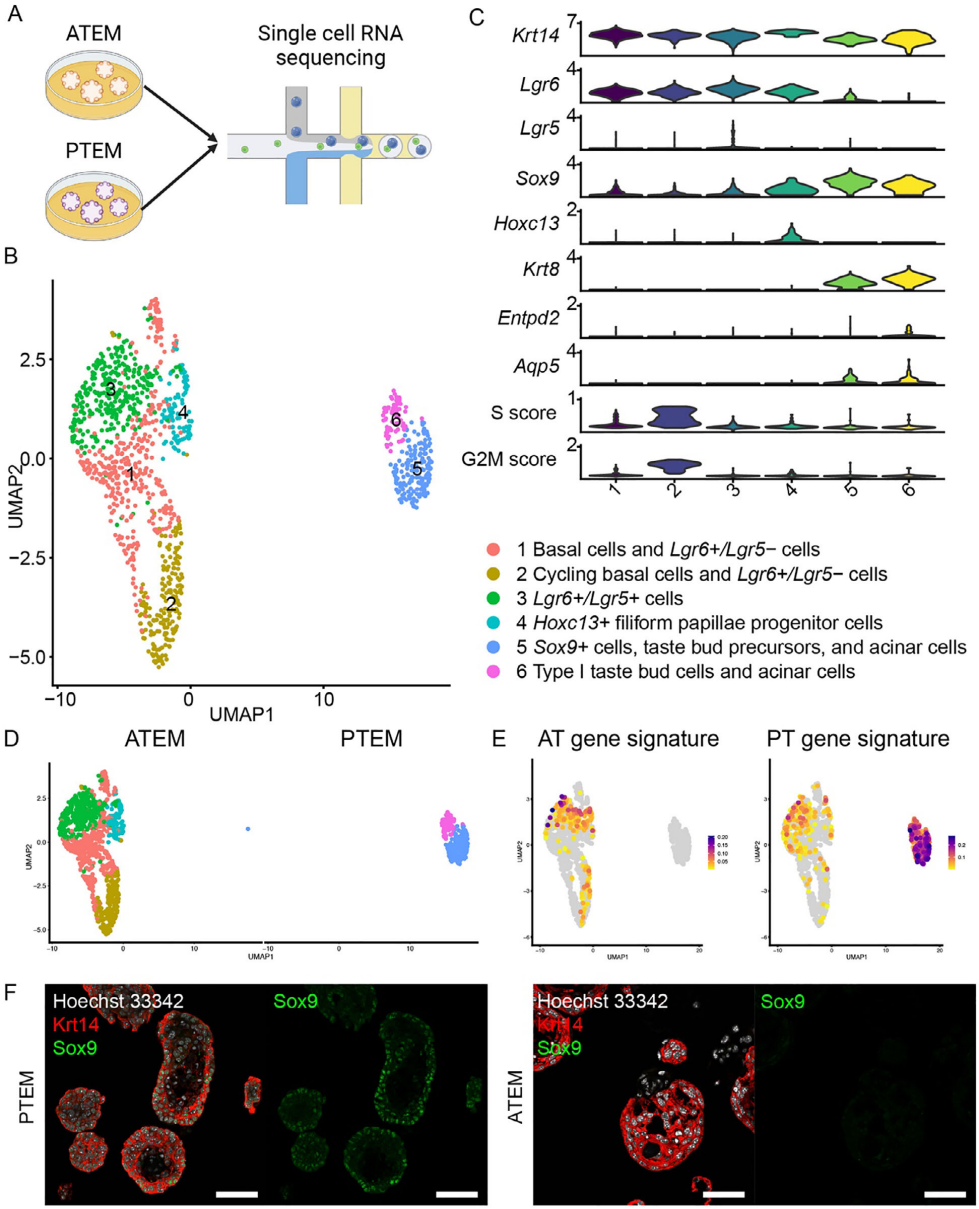
ATEM and PTEM retain region‐specific gene signatures at a single‐cell level. A) A workflow to process ATEM and PTEM for single‐cell RNA sequencing (scRNA‐seq). B) UMAP visualization of 6 clusters in ATEM and PTEM. Clusters were color‐coded. C) A violin plot showing the expression of known markers and cell cycle score for each cluster. D) UMAP split by tissue origins of each sample. E) UMAP showing AT and PT gene signature patterns. F) Representative fluorescent immunohistochemistry images showing expression of Sox9 (green) and Krt14 (red) in PTEM and ATEM. Hoechst 33 342 (white) marks the nuclei. Results from biological duplicates are shown. Scale bar, 50 µm. ATEM = anterior tongue organoids cultured in expansion medium; PTEM = posterior tongue organoids cultured in expansion medium.

To investigate whether ATEM preserves the region‐specific AT gene signature, we generated a 10X scRNA‐seq dataset of ATEM and PTEM (“organoid discovery dataset”) (**Figure** **3**A). After quality control, we obtained 1214 cells (ATEM: 979 cells, PTEM: 235 cells). We identified Hoxc13+ filiform papillae progenitor cells,^[^ ^26^ ^]^ cycling basal cells and basal cells (*Krt14+*), *Lgr6+/Lgr5‐* and *Lgr6+/Lgr5+* cells, *Sox9+* cells, type I taste bud cells (*Entpd2+*), and acinar cells (*Aqp5+*) (Figure 3B,C, Figure S6A,B, Supporting Information). ATEM consisted mostly of basal cells and *Lgr6+* cells, whereas PTEM contained a mixture of stem/progenitor cells and *Krt8+* lineage cells (Figure 3C). *Krt13+* keratinocytes were scarce and represented a different cluster from *Hoxc13+* filiform papillae cells (Figure S6B, Supporting Information). Yet, due to the limited number of *Hoxc13+* filiform papillae lineage cells in our scRNA‐seq data, *Krt13+* and *Hoxc13+* cells clustered in proximity (Figure S7A, Supporting Information). Therefore, to obtain higher resolution of the AT epithelial data, we exploited publicly accessible 10X scRNA‐seq data of Tabula Muris AT, which was reported to harbor filiform papillae lineage cells and various keratinocytes.^[^ ^26^ ^]^ Our analysis of integrated AT tissue and the Tabula Muris anterior tongue scRNA‐seq datasets (“tissue validation dataset”) confirmed inverse expression patterns between *Krt13* and *Hoxc13, Krt84*, and *Krt36* (Figure S7B,C, Supporting Information),^[^ ^26^ ^]^ in alignment with a previous study.^[^ ^27^ ^]^ We noted that UMAP clusters were strongly segregated by their tissue origins (Figure 3D). Importantly, the AT gene signature was more prominent in ATEM than in PTEM, whereas the PT gene signature was more prominent in PTEM than in ATEM (Figure 3E,F; Figure S8A, B, Supporting Information). For example, Sox9 expression was high in PTEM, as previously reported,^[^ ^10^ ^]^ but was almost absent in ATEM (Figure S8C, Supporting Information).

To further validate the regional identities of ATEM and PTEM (‘organoid validation dataset'), we performed scRNA‐seq on additional biological replicates [ATEM (n = 1) and PTEM (n = 2)] (Figure S9A, Supporting information). We obtained high‐quality cells from ATEM (n = 2121) and PTEM (n = 1917, 1770, respectively), including various epithelial cell types (Figure 3B) such as keratinocytes^[^ ^11^, ^21^ ^]^ and Ltf+ ductal cells (Figure S9B, Supporting Information).^[^ ^28^ ^]^ Again, the tissue origins of the organoids were strongly correlated with the segregation of UMAP clusters and the enrichment of region‐specific gene signatures (Figures S9C,D, and S10 Supporting information). These results demonstrated that ATEM contains various epithelial stem/progenitor cells that retain the AT gene signature.

### 2.3 Distinct Gene Regulatory Networks Control Regional and Cellular Identity

Transcription factors (TFs) determine cellular state by controlling the expression of their target genes (‘regulons’).^[^ ^29^ ^]^ To investigate how regional and cellular identity are shaped in dorsal tongue epithelium, we performed Single‐cell regulatory network inference and clustering (SCENIC) analysis and evaluated the Regulon Specificity Score (RSS). SCENIC is a computational method that analyzes scRNA‐seq data to predict regulons of TFs and their activities in individual cells.^[^ ^29^ ^]^ RSS is a metric showing how strongly a regulon is associated with a particular cell type or cluster.^[^ ^30^ ^]^

**Figure 4:**
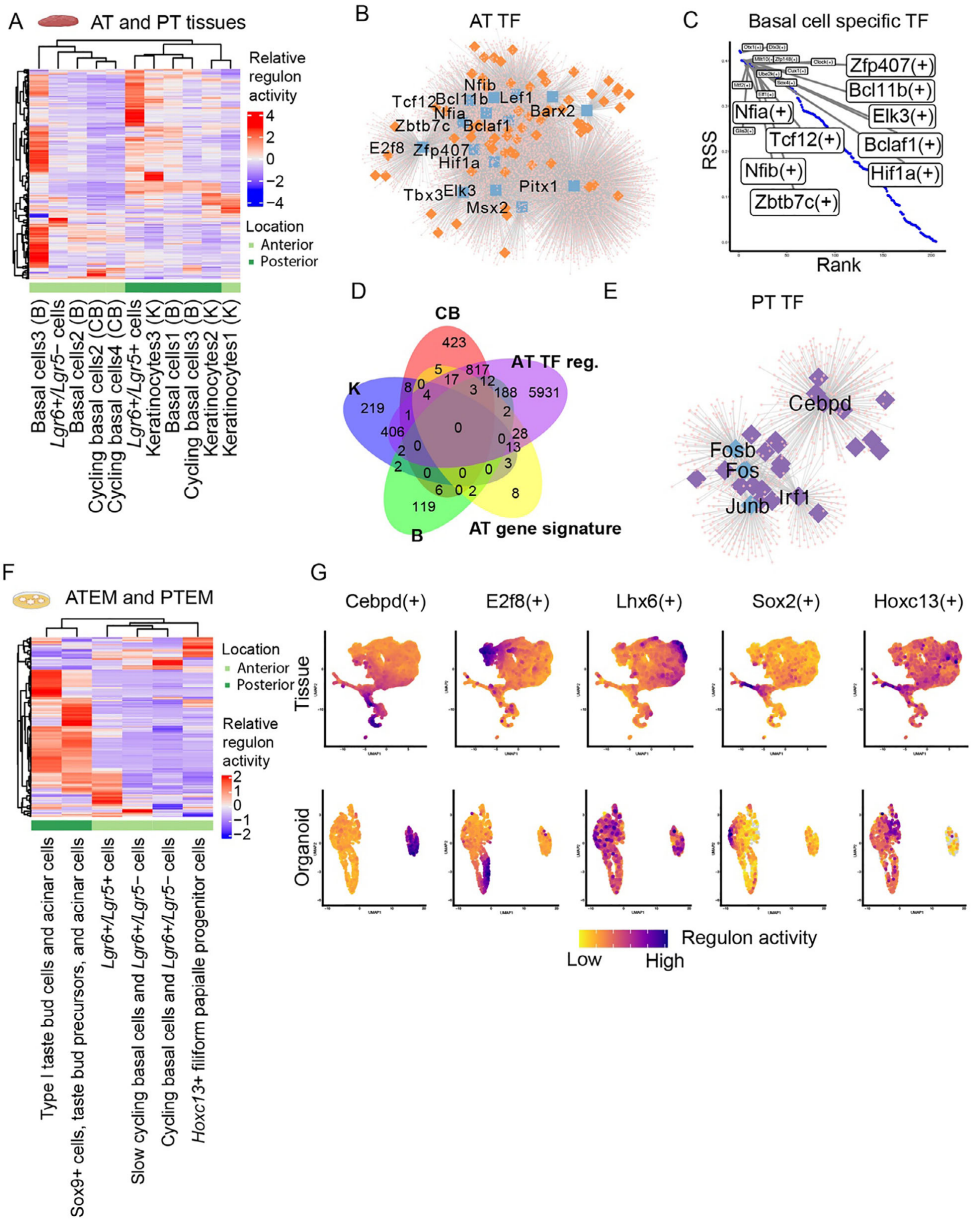
Distinct regulons control regional and cellular identity in the mouse dorsal tongue epithelium. A) A regulon activity matrix of AT and PT tissue scRNA‐seq dataset. Rows represent regulons and columns represent UMAP clusters. Tissue origins, cluster names, and their cell types (CB, cycling basal cells; B, basal cells; K, keratinocytes) are indicated below. Pearson correlation distance was used for hierarchical clustering. B) Network visualization of TFs that are predicted to regulate expression of >5% of genes in the AT gene signature (“AT TF”). TF (blue square with labels), genes in the AT gene signature (orange diamond), and other target genes of the TFs (pink dots) are indicated. C) An RSS plot showing the top 20 TFs that are specific to basal cells. Each dot indicates a TF. High RSS score (y‐axis) and low rank score (x‐axis) indicate high specificity of a TF to the cluster. AT TFs are highlighted. D) A Venn diagram showing a number of genes that are shared between 5 groups: markers for cycling basal cells (CB; n = 1296), basal cells (B; n = 336), keratinocytes (K; *n* = 658), AT gene signatures (*n* = 85), and regulons of AT TFs (*n* = 7424). Each oval represents one of the 5 groups. A number of shared genes is indicated where ovals overlap, and a number of genes unique to each group is indicated where ovals don't overlap. E) Network visualization of TFs that are predicted to regulate expression of >5% of genes in the PT gene signature (“PT TF”). TF (blue square with labels), genes in the PT gene signature (purple diamond), and target genes of the TFs (pink dots) are indicated. G) A regulon activity matrix of ATEM and PTEM scRNA‐seq data. Rows represent regulons and columns represent UMAP clusters. Pearson correlation distance was used for hierarchical clustering. I) UMAP plots showing examples of region‐specific and cell‐type‐specific regulon activities in tissues and organoids.

We applied both SCENIC and RSS analysis to the tissue (AT and PT) and organoid (ATEM and PTEM) scRNA‐seq datasets. In tissue, we found that most AT‐enriched UMAP clusters and PT‐enriched UMAP clusters were grouped separately based on the activity of their TF regulons. This indicates that different gene regulatory networks are active in AT and PT tissues (**Figure** **4**A).

Next, we interrogated which TFs could be responsible for these regional differences. In AT tissue, TFs such as *E2f8*, *Bcl11b*,^[^ ^31^ ^]^ and *Pitx1* appeared to play key roles (Figure 4B). Interestingly, these TFs (“AT TFs”) were particularly active in cycling basal cells (i.e., *E2f8, Pitx1*), basal cells (i.e., *Bcl11b*), and keratinocytes (i.e., *Pitx1*) among all epithelial cell types, as shown by high RSS values (Figure 4C; Figure S7, Supporting Information). To further explore this, we examined marker genes that are specific to cycling basal cells (n = 1296), basal cells (n = 336), and keratinocytes (n = 658) (Figure 4D). A large portion of these marker genes was shared by the regulons of AT TFs [cycling basal cells (854/1296; 65.9%), basal cells (207/336; 61.6%), and keratinocytes (426/658; 64.7%)] (Figure 4D). Additionally, gene set enrichment analysis (GSEA) demonstrated that the marker genes for these cell types are involved in various biological processes (e.g., chromosome segregation and Wnt signaling pathway) that are shared by the regulons of AT TFs (Figure S11, Supporting Information). This indicated that AT TFs may determine both regional (AT) and cellular (cycling basal cells, basal cells, and keratinocytes) identity.

In the PT tissue, different TFs were prominent. These TFs included *Cebpd* and the activator protein 1 (AP‐1) family genes (*Fos*, *Fosb*, and *Junb*) (Figure 4E) (“PT TFs”). The AP‐1 family TFs have previously been implicated in cell proliferation and maintenance of taste bud structures in the PT tissue.^[^ ^32^, ^33^ ^]^ In our analysis, these TFs were not strongly linked to any particular epithelial cell type based on RSS (Figure S12A, Supporting Information). GSEA showed that regulons of PT TFs are involved in the regulation of epithelial cell proliferation (Figure S11, Supporting Information), indicating their distinct functions compared to AT TFs.

We also found cell‐type‐specific TFs (Figure S12A, Supporting Information). For example, *Sox2*, *Foxa2*, and their respective regulons were highly active in *Lgr6+/Lgr5+* cells. These cells also expressed *Gli1* and *Ptch1*, implying a critical involvement in filiform and fungiform papillae homeostasis (Figure S12B, Supporting Information) as reported previously.^[^ ^18^, ^19^, ^21^, ^34^, ^35^ ^]^ In contrast, TFs such as *Lhx6* and *Klf4* were highly active in *Lgr6+/Lgr5‐* cells (Figure S12A, supporting information).

Importantly, organoids showed similar patterns by which clusters were grouped separately through region‐based activity of regulons. Organoids (ATEM and PTEM) partially retained region‐ and cell type‐specific gene regulatory networks observed in the tissues (Figure 4I; Figures S12A and S13A,B, Supporting Information). For example, *Hoxc13+* filiform papillae progenitor cells were driven by *Hoxc13*, which we confirmed in the tissue validation dataset (Figure 4G; Figure S13A, C, Supporting Information). Overall, our results suggested that specific TFs and their regulons may shape both the regional identity (AT and PT) and cellular diversity of the dorsal tongue epithelium.

### 2.4 Intercellular Communication Networks in Anterior Tongue Epithelium

**Figure 5:**
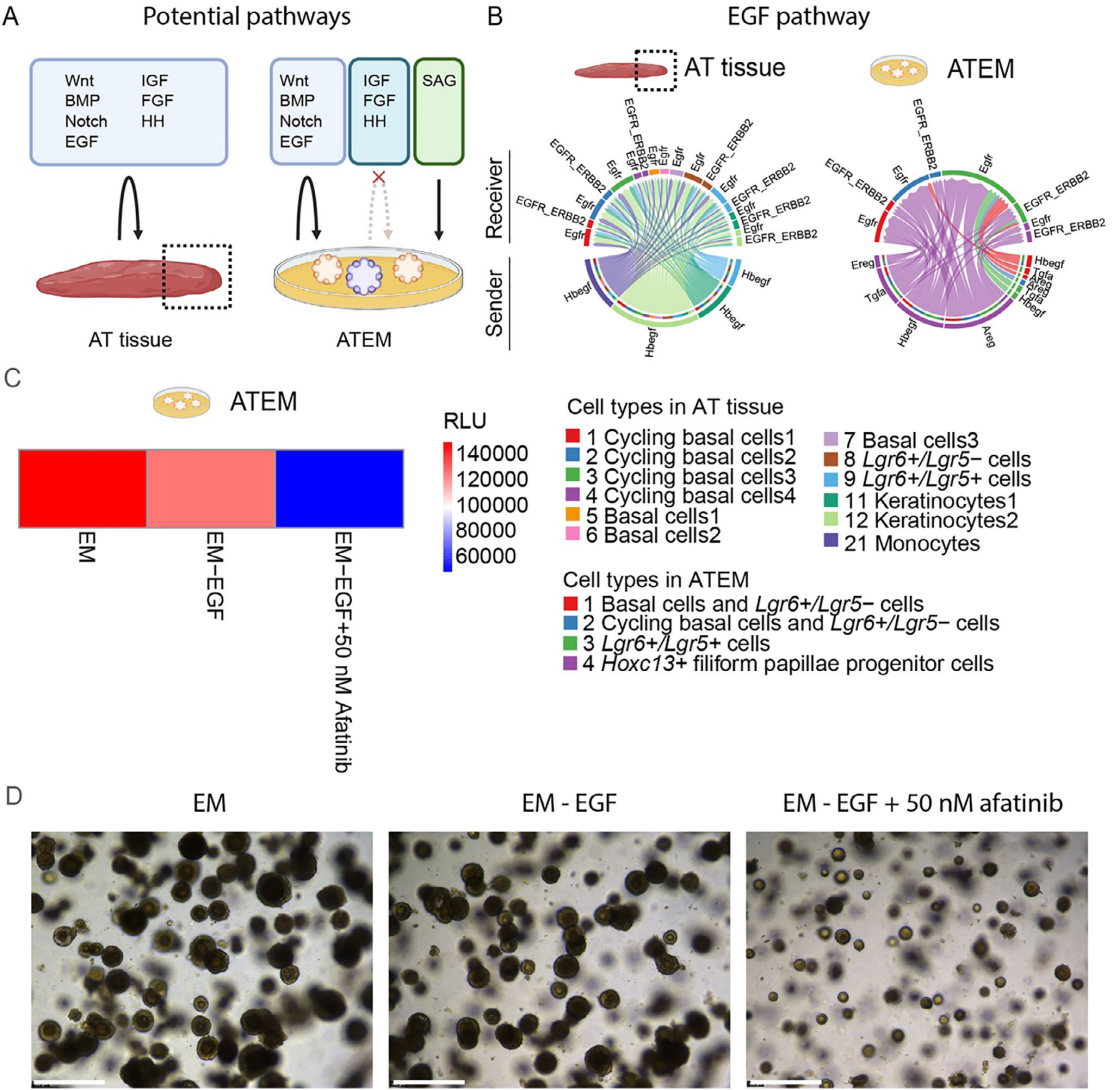
Organoid‐based functional validation of computationally predicted intercellular signaling pathways in AT tissue. A) A graphic summary showing potential receptor‐ligand interactions predicted by CellChat analysis in the AT tissue and ATEM scRNA‐seq data. Ligands for Wnt, BMP, Notch, and EGF signaling pathways are mainly produced by and recognized by epithelial cells in both tissue and organoids. In tissue, ligands for IGF, HH, and FGF signaling pathways are produced by taste bud cells or fibroblasts, cell types of which are absent in ATEM. In ATEM, HH signaling is exogenously activated due to the addition of SAG to the culture medium. B) Chord diagrams showing “senders” and “receivers” of the EGF signaling pathway in AT tissue and ATEM. Cell types are color‐coded. C) AT organoids were cultured in the indicated medium composition for 7 days. Cell viability was measured using CellTiterGlo 3D. RLU, relative luminescence unit. Results from technical triplicates of biological duplicates are shown. D) Representative bright‐field images of AT organoids, which were cultured in the indicated medium compositions for 7 days. Results from technical triplicates of biological duplicates are shown. Scale bar, 500 µm.

To better understand intercellular communication networks in the AT epithelium, we applied CellChat analysis^[^ ^36^ ^]^ to our AT tissue (including all cell types) and ATEM scRNA‐seq data containing all cell types (**Figure** **5**A). In tissue, we observed strong interactions between epithelial cells and between epithelial and stromal cells (fibroblasts and endothelial cells) (Figure S14, Supporting Information). Autocrine and/or paracrine Wnt, Notch, BMP, and EGF pathways were present mainly in epithelial cells and thus well captured in both AT tissue and ATEM (Figure 5A,B; Figure S15A,B, Supporting Information). These pathways have been implicated in tongue epithelium homeostasis.^[^ ^23^, ^37^, ^38^, ^39^ ^]^ We noted signaling pathways that are unique to the tissue and not seen in ATEM, likely due to the absence of certain cell types in ATEM (Figure 5A; Figure S15C, Supporting Information). In tissue, taste bud cells were a major provider for Igf1 and Shh, and fibroblasts were a major provider for FGFs, in alignment with previous findings.^[^ ^20^, ^40^, ^41^, ^42^, ^43^ ^]^ *Lgr6+/Lgr5+* cells were a major receiver of Shh, confirming an important role of Shh in maintaining taste bud precursors.^[^ ^18^, ^20^, ^42^ ^]^ In ATEM, we observed strong *Sox2* regulon activity and *Gli1* and *Ptch1* expression in *Lgr6+/Lgr5+* cells (Figure 4I; Figure S13B, Supporting Information) due to exogenous activation of Shh signaling by SAG.^[^ ^14^ ^]^

To test whether Wnt, Notch, BMP, EGF, IGF1, and FGF signaling pathways are involved in cell proliferation, we withdrew and/or added growth factors and chemical compounds in ATEM to block or activate these pathways (Figure S15D, Supporting Information). Interestingly, EGF withdrawal combined with the addition of 50 nM afatinib, a pan‐ERBB inhibitor, dramatically inhibited cell growth. We reasoned that, if ATEM produces its own ligands to activate the EGF pathway, as predicted by CellChat analysis (Figure 5B), withdrawal of EGF from the expansion medium would not impact cell proliferation of ATEM. Indeed, EGF withdrawal alone did not impair cell viability in the AT organoids, whereas EGF withdrawal combined with the addition of afatinib did (Figure 5C,D). Together, these results illustrated that ATEM captures the epithelial cell‐to‐epithelial cell interactions of AT tissue (Figure 5D).

### 2.5 Anterior Tongue Organoids Can be Differentiated into Various Epithelial Cells

**Figure 6:**
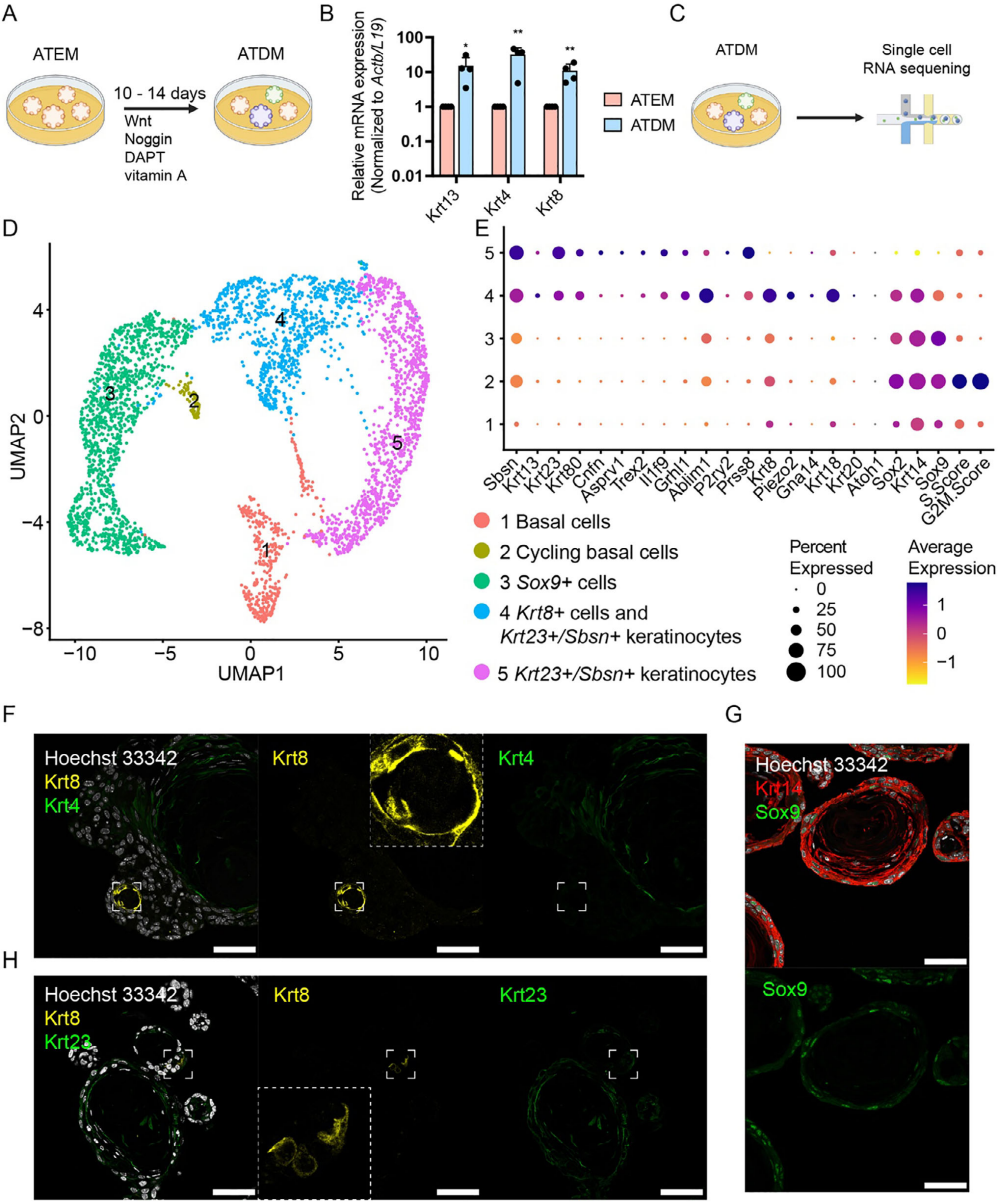
Modeling anterior tongue epithelial cell differentiation using organoids. A) A workflow to differentiate anterior tongue (AT) organoids. AT organoids were initially cultured in expansion medium (ATEM), and subsequently for 10 to 14 days in the differentiation medium (ATDM), where R‐spondin 1, SAG, A83‐01 were removed and DAPT and vitamin A were added. B) Quantitative PCR comparing expression of markers for epithelial cell types in ATEM and ATDM (two‐tailed *t*‐test: ^*^ *p* < 0.05; ^**^ *p* < 0.01). Results from technical triplicates of biological quadruplicates are shown. C) A workflow to process ATDM for single‐cell RNA sequencing. D) UMAP visualization of 5 clusters in ATDM. Cell types are color‐coded. E) A dot plot showing expression of cell type markers and cell cycle scores in ATDM. F) Representative fluorescent immunohistochemistry images showing expression of Krt8 (yellow) and Krt4 (green) in ATDM. Krt8+ cells are zoomed in. G) Representative fluorescent immunohistochemistry images showing expression of Sox9 (green) and Krt14 (red) in ATDM. H) Representative fluorescent immunohistochemistry images showing expression of Krt8 (yellow) and Krt23 (green) in ATDM. Krt8+ cells are zoomed in. Hoechst 33 342 marks the nuclei. F,G,H) Hoechst 33 342 marks the nuclei. Results from biological duplicates are shown. Scale bar, 50 µm.

We next investigated whether stem/progenitor‐like ATEM can differentiate into more specialized epithelial cells, including *Krt8+* cells and/or *Krt13+* cells. To differentiate ATEM, we removed R‐spondin 1, EGF, SAG, and A83‐01 from the expansion medium and added the γ‐secretase inhibitor DAPT and vitamin A to the medium. DAPT and vitamin A were included because they can induce taste bud cell or organoid differentiation.^[^ ^44^, ^45^ ^]^ The AT organoids were exposed to the differentiation medium (termed “ATDM”) for 2 weeks (**Figure** **6**A). Compared to ATEM, ATDM showed increased expression of *Krt4*, *Krt13* (general markers for keratinocyte), and *Krt8* (Figure 6B; Figure S16A, Supporting Information).

To further explore the cellular heterogeneity of ATDM, we performed 10X scRNA‐seq on ATDM (Figure 6C) and obtained 3696 high‐quality cells (Figure 6D). We identified *Krt8+* cells, *Krt23+/Sbsn*+ keratinocytes, *Sox9*+ cells, cycling basal cells, and basal cells (Figure 6F,G,H; Figure S16B, Supporting Information). Unexpectedly, *Krt8*+ cells showed high expression of *Piezo2* (Figure 6E; Figure S17, Supporting Information), a mechanosensing ion‐evoked channel that is crucial to tactile response, pain response, and proprioception.^[^ ^46^, ^47^, ^48^ ^]^ *Piezo2* is commonly expressed in Merkel cells, which are found in the hair follicle and oral cavity, and generally marked by *Krt8*, *Krt18, Sox2, Krt20*, and/or *Atoh1*.^[^ ^5^, ^6^, ^49^, ^50^, ^51^ ^]^ Indeed, *Krt8+/Piezo2+* cells expressed *Krt18* and *Sox2*, yet lacked *Krt20* and *Atoh1* expression (Figure 6E). Furthermore, we identified *Krt8*+ cells expressing taste bud cell markers such as *Gna14* (Figure S17, Supporting Information). Expression of other mature taste bud cell markers (*Entpd2*, *Gnat3*, *Chga*, and *Pkd2l1*) and mature filiform papillae cell markers (*Krt84*, *Krt36*) was generally not very high (Figure S17, Supporting Information). *Krt23+/Sbsn+* keratinocytes^[^ ^26^, ^52^ ^]^ expressed many skin epidermis‐related markers (*Krt80*, *Cnfn*, *Asprv1*, *Trex2*, *Ilf19*, *Prss8, Grhl1, and Ablim1*).^[^ ^53^, ^54^, ^55^, ^56^, ^57^, ^58^, ^59^, ^60^ ^]^ Corroborating this observation, the marker genes and the gene signature of *Krt23+/Sbsn+* keratinocytes were specific to “differentiated”, “suprabasal differentiating”, “suprabasal differentiated”, and “filiform papillae differentiated” clusters in the tissue validation dataset (Figure S18A, Supporting Information). Furthermore, Krt23+ cells were mostly localized to the suprabasal interpapillary region in the AT tissue (Figure S18B, Supporting Information). Together, these results demonstrated cellular heterogeneity of ATDM.

### 2.6 Gene Regulatory Network Analysis Identifies Transcription Factors Associated with *Krt8+* and *Krt23+*/*Sbsn*+ Cell Differentiation

**Figure 7:**
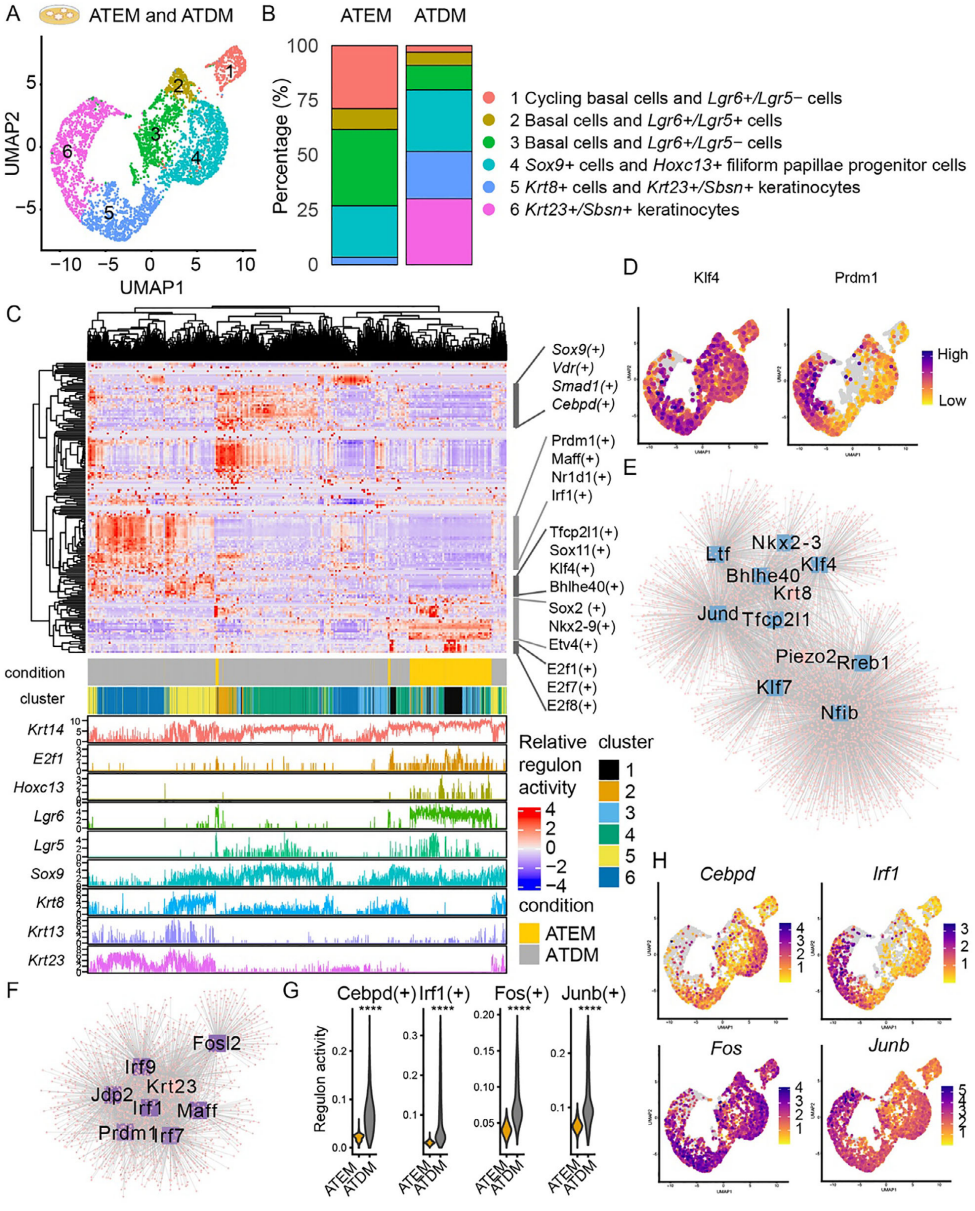
Gene regulatory network analysis identifies transcription factors associated with *Krt8+* cells and *Krt23+/Sbsn+* keratinocytes. A) UMAP showing integrated single‐cell RNA sequencing data of ATEM and ATDM. B) A bar plot showing the proportion of clusters in ATEM and ATDM. A, B) Clusters are color‐coded. C) A heatmap plot showing the relative activity of regulons and normalized expression of cell type markers in ATEM and ATDM. Each column represents a single cell, and each row represents a regulon. Cells and regulons were clustered using Pearson correlation of relative regulon activity. Cluster‐specific regulons that were identified using RSS are indicated (right). D) UMAP plots showing expression of *Tfcp2l1* and *Prdm1*. E) Network visualization of potential transcription factors (blue square) that regulate *Krt8* and *Piezo2* expression. Each dot (pink) indicates a predicted target gene of the transcription factors (blue). F) Network visualization of potential transcription factors (purple square) that regulate *Krt23* expression. Each dot (pink) indicates a predicted target gene of the transcription factors (purple). G) Regulon activity of *Cebpd*, *Irf1*, *Fos*, and *Junb* in ATEM and ATDM. (Mann‐Whitney U‐test: ^***^ *p* < 2.2e‐16). H) UMAP plots showing expression of *Cebpd*, *Irf1*, *Fos*, and *Junb*. ATEM = anterior tongue organoids cultured in expansion medium; ATDM = anterior tongue organoids cultured in differentiation medium.

To investigate cellular and molecular changes associated with epithelial cell differentiation in the AT epithelium, we integrated scRNA‐seq datasets of ATEM and ATDM (**Figure** **7**A). We noted that the percentage of cycling basal cells, basal cells, and *Lgr6+/Lgr5‐* cells was dramatically decreased while *Krt8+* and *Krt23+/Sbsn+* keratinocytes were increased (Figure 7B,C).

Next, to investigate TFs that are associated with epithelial cell differentiation, we performed SCENIC analysis on the integrated dataset. In *Krt8*+ cells, TFs such as *Klf4* appeared to play an important role (Figure 7C,D,E; Figure S19, Supporting Information). Additionally, TFs including *Klf7*, *Rreb1*, *Nfib, Nkx2‐3, and Tfcp2l1* were predicted to control *Piezo2+* expression (Figure 7E). This indicated that *Krt8+* cells may require extra transcription factors to acquire the Merkel‐like cell fate.

On the other hand, TFs including *Prdm1*, *Maff* were highly active in *Krt23+/Sbsn*+ keratinocytes (Figure 7C,D,F) along with *Pitx1* (Figure S12A, Supporting Information). *Prdm1* and *Maff* are involved in keratinocyte differentiation in skin.^[^ ^53^, ^55^ ^]^ Surprisingly, *Cebpd* and *Irf1*, which we identified as PT TFs (Figure 4F), were highly active in *Sox9*+ and *Krt23+/Sbsn*+ cells, respectively, suggesting putative roles of the PT TFs in keratinocyte differentiation in the anterior tongue (Figure 7C; Figure S19, Supporting Information). Indeed, regulon activity of PT TFs, including *Cebpd*, *Irf1*, *Fos*, and *Junb*, was significantly increased in ATEM compared to ATDM (Figure 7G). Expression of the PT TFs was high in *Sox9*+ (*Cebpd*) and *Krt23+/Sbsn+* cells (*Irf1*, *Junb*), respectively (Figure 7H). *Fos* was universally expressed in multiple clusters, while *Fosb* regulon activity wasn't detected in this dataset.

Similarly, regulon activities of *Prdm1* and/or *Maff* were specifically enriched in “differentiated”, “suprabasal differentiating”, “suprabasal differentiated”, and “filiform papillae differentiated” clusters in the tissue validation dataset (Figure S20A, Supporting Information). Among these, “suprabasal differentiating” and “filiform papillae differentiated” clusters showed increased activity and/or expression of *Sox9*, *Cebpd*, *Fos*, and *Junb* (Figure S20B, Supporting Information). This indicated that in vitro differentiation models “suprabasal differentiating” and “filiform papillae differentiated”. Together, our results demonstrate that *Krt8*+ cells and *Krt23+/Sbsn+* cells harbor distinct gene regulatory networks.

**Figure 8:**
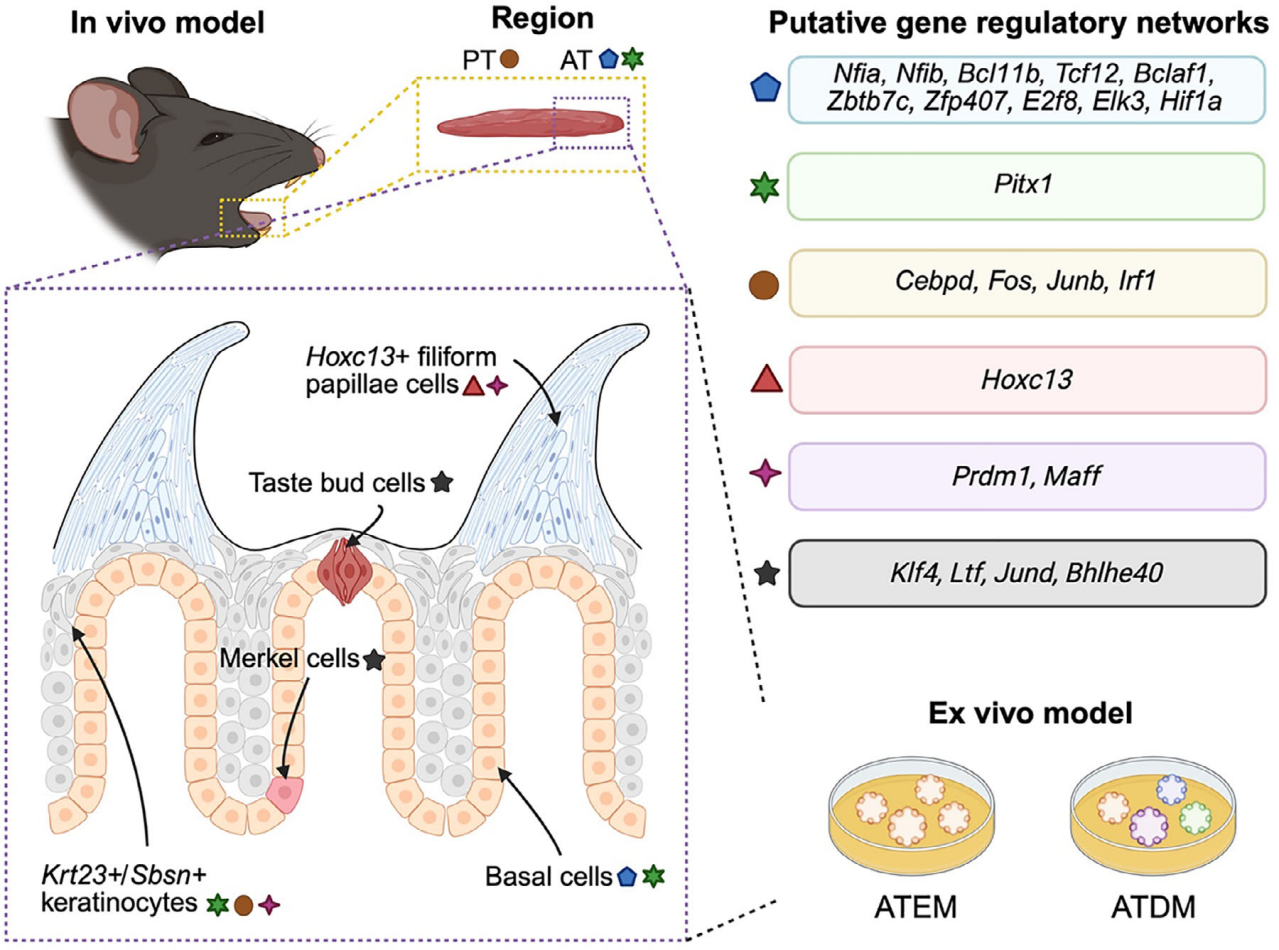
Graphic model of gene regulatory networks in dorsal tongue epithelial tissues and organoids. In the PT tissue, *Cebpd, Fos, Junb, and Irf1* and their regulons are enriched. In the AT tissue, *Nfia, Nfib, Bcl11b, Tcf12, Bclaf1, Zbtb7c, Zfp407, E2f8, Elk3, Hif1a*, and *Pitx1 and their regulons* are enriched, showing that AT and PT epithelia have different gene regulatory networks. These AT‐associated TFs also shape the cellular identity of basal cells and *Krt23+/Sbsn+* keratinocytes in the AT tissue. *Prdm1* and *Maff* are active in both *Krt23+/Sbsn+* keratinocytes and Hoxc13+ filiform papillae cells, whereas *Hoxc13* activity is specific to filiform papillae lineage cells. *Klf4, Ltf, Jund*, and *Bhlhe40* control *Krt8*+ cell fates. The homeostasis and underlying gene regulatory networks of anterior dorsal tongue epithelium can be modeled by ATEM and ATDM.

In summary, our results implied that AT epithelium is driven by basal cell/keratinocyte lineage‐associated TFs, whereas PT epithelium is driven by cell migration‐ and proliferation‐associated TFs (**Figure** **8**). During epithelial cell differentiation, *Krt8*+ cells and *Krt23+/Sbsn*+ keratinocytes follow distinct paths by which *Krt23+/Sbsn*+ keratinocytes are influenced by PT‐associated TFs (Figure 8).

## 3 Discussion

In this study, we have optimized a culturing protocol for anterior tongue (AT) organoids. AT organoids in expansion medium, ATEM, retain the region‐specific gene expression signature of AT tissue. Furthermore, both ATEM and ATDM harbor various stem/progenitor cell types (basal cells, *Lgr6+/Lgr5‐* and *Lgr6+/Lgr5+* cells, *Hoxc13+* filiform papillae progenitor cells) and specialized epithelial cell types (taste bud cells, keratinocytes, and Merkel‐like cells).

Previous studies have noted that cellular composition (e.g., taste bud cell subtypes) and cellular responses to perturbations β‐catenin and Shh) may differ between anterior and posterior tongue epithelium.^[^ ^61^ ^]^ Most scRNA‐seq studies^[^ ^26^, ^62^, ^63^, ^64^, ^65^, ^66^ ^]^ have focused on PT tissue, or on specific cell types (including Type II taste bud cells or immune cells) in AT tissue. Therefore, the transcriptional complexity of anterior tongue epithelium has remained unclear. Our study provides an unbiased scRNA‐seq dataset of mouse dorsal tongue tissues and organoids. Using scRNA‐seq data and organoid models, we demonstrate that AT and PT epithelial cells harbor region‐specific and cell type‐specific gene regulatory networks. AT TFs are associated with basal cells and keratinocytes, which may explain a paucity of taste bud cells and abundance of keratinocytes in the anterior tongue compared to the posterior tongue.^[^ ^26^, ^66^ ^]^ *Cebpd, Fos, and Jun* have been implicated in aging,^[^ ^66^ ^]^ cell proliferation, and maintenance of taste bud structures in the PT tissue.^[^ ^32^, ^33^ ^]^ Interestingly, PT TFs were enriched in *Krt23+/Sbsn+* keratinocytes, highlighting their differential roles in the anterior tongue homeostasis. We demonstrated that AT and PT organoids capture these region‐specific gene signatures. Similarly, mouse intestinal organoids recapitulate location‐specific functions that are epigenetically programmed in adult stem cells.^[^ ^67^ ^]^ How this region‐specific gene regulatory network arises during development remains to be investigated.

A previous study has shown that mouse tongue organoids harboring stratum corneum can be generated using R‐spondin, Noggin, and EGF.^[^ ^13^ ^]^ Yet, long‐term expansion and cellular complexity of these organoids were not demonstrated, for which we have now developed an optimized medium. Of note, it is not indicated which region of the tongue epithelium was used to initiate the organoid cultures. In this study, we demonstrate that Hedgehog pathway activation (SAG) and TGF‐β pathway inhibition (A83‐01) are critical for long‐term passaging of ATEM. We noted that the morphology and cellular composition of ATEM varied among the lines (Figure 1E,B; Figures S1B and S9, Supporting Information). *Hoxc13*+ filiform papillae progenitors, *Lgr6+/Lgr5‐*, and *Lgr6+/Lgr5+* cells with high *Ptch1* and *Gli1* expression and Sox2 regulon activities are maintained in ATEM. Accordingly, in vivo studies have demonstrated that the Shh – Ptch1 – Gli1 signaling axis is important for the maintenance of CvP, FoP, FP, and filiform papillae.^[^ ^16^, ^17^, ^18^, ^19^, ^20^, ^35^ ^]^ Particularly, the Gli1+ cells were shown to give rise to filiform and fungiform papillae.^[^ ^19^ ^]^ The identity of molecular cues that will differentiate the Gli1+ cells into the Shh+ precursors and mature taste bud cells remains to be investigated.

In contrast to ATEM, PTEM can grow multiple passages in their expansion medium without SAG and A83‐01,^[^ ^4^, ^10^, ^11^, ^21^, ^45^, ^68^ ^]^ and the effects of Shh modulators on these organoids remain unclear.^[^ ^4^, ^21^, ^68^ ^]^ These findings and our results suggest that Shh signaling exerts region‐specific effects.^[^ ^42^ ^]^ We observed that IGF1 and FGFs, which were predicted to be received by epithelial cells in the anterior tongue, are dispensable for the passaging of ATEM. Furthermore, the addition of IGF1, FGF1, and FGF7 did not improve the long‐term passaging capacity of ATEM. In line with this, a previous study demonstrated that genetic ablation of the *Igf1* receptor does not impact tongue tissue homeostasis despite its high expression in taste buds.^[^ ^41^ ^]^ A recent study demonstrated that FGFs can increase organoid‐forming efficiency from ventral and dorsal oral mucosa tissues over a period of 7 days,^[^ ^43^ ^]^ which may suggest that FGFs are involved in stress recovery.

We demonstrate that Wnt, Noggin, DAPT, and vitamin A can drive differentiation of ATEM into Krt8+ cells (Merkel‐like cells and *Gna14+* taste bud cells) and *Krt23+/Sbsn+* keratinocytes. Wnt activation, BMP pathway inhibition by Noggin, and Notch inhibition by DAPT promoted taste bud cell lineage specification in PTEM,^[^ ^45^, ^68^ ^]^ suggesting that signaling pathways involved in taste bud cell differentiation are conserved between the anterior and posterior tongue.

Embryonic development of Merkel cells in the hair follicle is controlled by Atoh1, Sox2, and Isl1.^[^ ^51^ ^]^ In the adult hair follicle, the Merkel cell progenitor is marked by Atoh1.^[^ ^69^ ^]^ Merkel cells are sparse in the filiform and fungiform papillae of rodents and humans, and their physiological functions and origins remain unclear.^[^ ^5^, ^6^, ^70^ ^]^ Interestingly, we observe expression of *Sox2* and *Isl1* in adult AT tissue. Furthermore, we note that ATDM contains Merkel‐like cells expressing *Piezo2, Krt8, Krt18*, and *Sox2*, but not *Atoh1* or *Krt20*. The fact that these cells lack *Atoh1* and *Krt20* expression could be explained by i) their immature cell state,^[^ ^49^ ^]^ ii) different transcription factors governing Merkel cell specification in the dorsal tongue than in the skin, or iii) different progenitors giving rise to Merkel cells in the dorsal tongue. Notably, skin injury stimulated *Atoh1‐/Gli1+* adult skin progenitor cells to produce Merkel cells.^[^ ^71^ ^]^ This raises an interesting possibility that ATDM mimics a wound‐healing process where *Lgr6*+/*Lgr5+/Gli1+* cells give rise to *Atoh1*‐ Merkel‐like cells. The physiological relevance of Merkel‐like cells and *Sox9*+ cells still remains to be investigated.

We would like to mention caveats of the current study: our scRNA‐seq data of tissue and PTEM lack Merkel cells and Lgr5+ cells, respectively, likely due to the sparsity and fragility of these cell types and the generally low level of Lgr5 expression in stem cells.^[^ ^72^ ^]^ The current protocol for differentiating ATEM does not fully capture mature taste bud cells or mature filiform papillae cells, and further optimization will be required to enrich these populations. In follow‐up studies, CRISPR screening in organoids or the generation of transgenic mouse models can be used to validate the predicted roles of transcription factors identified by in silico analysis in regional or cellular identity decisions. In conclusion, we have generated AT organoids that can be used to explore the biology of the anterior and posterior epithelium of the dorsal tongue.

## 4 Experimental Section

### Animal Studies

All animal experiments were performed under the guidelines and approval of the animal welfare committee of the Netherlands Cancer Institute (34.1.11286) and Hubrecht Institute (KNAW) (AVD 80 100 2020 9924).

### Organoid Culture

PTEMs were generated as previously reported.^[^ ^4^, ^10^ ^]^ In brief, mouse circumvallate papillae and foliate papillae tissues were collected using tweezers and finely chopped before being embedded in Cultrex Basement Membrane Extract (BME). PTEM were cultured in medium containing Advanced DMEM‐F12 (Gibco, Waltham, MA, USA), 1X B27 without vitamin A (Gibco), 1X Pen/Strep (Gibco), 10 mM HEPES (Gibco), 1.25 mM N‐acetylcysteine (Sigma‐Aldrich, MA, USA), 1X Glutamax (Gibco), 50 ng mL^−1^ EGF (AF‐100‐15; PeproTech, NJ, USA), 10% noggin conditioned medium, and 10% R‐spondin 1 conditioned medium.^[^ ^4^, ^10^ ^]^ PTEM were passaged at ≈1:4 split ratios every two weeks.

To generate ATEM, ≈2 mm from the tip of the mouse tongue tissue was cut, minced, and embedded in BME. ATEM were cultured in expansion medium containing Advanced DMEM‐F12 (Gibco), 1X B27 without vitamin A (Gibco), 1X Pen/Strep (Gibco), 10 mm HEPES (Gibco), 1.25 mm N‐acetylcysteine (Sigma‐aldrich), 1X Glutamax (Gibco), 50 ng mL^−1^ EGF (Peprotech), 10% noggin‐conditioned medium, 10% R‐spondin 1‐conditioned medium, 0.5 nm Wnt‐surrogate, 30 nm SAG (Selleckchem, TX, USA), and 500 nm A83‐01 (Tocris, Bristol, UK). For passaging, organoids were dissociated in TrypLE containing 10 µm Y‐27538 (Abmole BioScience, TX, USA) at 37 °C for up to 7 min before being pipetted using a 1000 uL tip for up to 30 s, and subsequently embedded in BME. In the initial 3 passages, organoids were split at 1:2 ratios every 2 to 3 weeks and cultured in Y‐27632. Afterward, ATEM were passaged at ≈1:4 split ratios every two weeks. To test the effect of SAG and A83‐01 on establishing ATEM, the tip of the tongue was minced and cultured in the presence or absence of SAG and A83‐01 until they stopped growing (biological triplicates).

To assess whether Wnt, Notch, BMP, EGF, IGF1, and FGF signaling pathways affect epithelial cell proliferation, ATEM were cultured for up to two months in expansion medium with the following reagents withdrawn and/or supplemented: Wnt‐surrogate, 3 µm IWP2 (Tocris), R‐spondin 1 conditioned medium, 10 µm DAPT (Tocris), EGF, 50 nm afatinib (Selleckchem), noggin‐conditioned medium, 100 ng mL^−1^ IGF1 (Peprotech), 20 ng mL^−1^ FGF1 (Peprotech), 20 ng mL^−1^ FGF7 (Peprotech). To test whether ATEM produces its own EGF pathway ligands, the organoids were seeded on 48‐well plates (technical triplicates and biological duplicates) and cultured for 7 days in expansion medium, expansion medium without EGF, and expansion medium without EGF supplemented with 50 nm afatinib. Then, the Cell Titer Glo 3D (G9683; Promega, Madison, WI, USA) cell viability assay was performed.

To differentiate ATEM, organoids were first dissociated in TrypLE + 10 µm Y‐27538 for up to 10 min at 37 °C. Single cells and small organoid fragments were collected using a 70 µm strainer, and subsequently embedded in BME and grown in ATEM medium for 3–4 days. Then, the organoids were cultured in differentiation medium containing Advanced DMEM‐F12 (Gibco), 1X B27 with vitamin A (Gibco), 1X Pen/Strep (Gibco), 10 mm HEPES (Gibco), 1.25 mm N‐acetylcysteine (Sigma‐Aldrich), 1X Glutamax (Gibco), 10% noggin‐conditioned medium, 0.5 nm Wnt‐surrogate, and 10 µm DAPT.

### RNA Extraction and Quantitative PCR

RNA was isolated from organoids using the RNeasy kit (74 104; Qiagen, Hilden, Germany). cDNA was synthesized using SuperScript III Reverse Transcriptase (ThermoFisher Scientific) following the manufacturer's instructions.

Quantitative PCR was performed using iQ SYBR Green Supermix (Bio‐Rad) following the manufacturer's instructions. The primer list is included in Table S1 (Supporting Information).

### Immunofluorescence

Tongue organoids were fixed in fresh 4% PFA at room temperature for 30 min, and tissues were fixed in 10% neutral buffered formalin at 4 °C overnight before being dehydrated and embedded in paraffin. The paraffin blocks were cut into 4 µm slides using the Rotary Microtome Microm HM355S (Thermo Fisher Scientific).

Fluorescent immunohistochemistry was performed using the following antibodies or dye: Krt8 (TROMA‐I; DSHB), Krt14 (906 004; Biolegend, CA, USA), Sox2 (AF2018; R&D systems, MN, USA), Krt4 (PA5‐102642; Invitrogen, MA, USA), Sox9 (AB5535; Sigma‐Aldrich), Pitx2 (ab221142; Abcam, Cambridge, UK), Krt23 (24049‐1‐AP; Proteintech, IL, USA), Hoechst 33 342 (H3570, Thermo Fisher Scientific), Donkey anti‐Rat IgG (H+L) Highly Cross‐Adsorbed Secondary Antibody, Alexa Fluor 568 (A78946; Invitrogen), Donkey anti‐Chicken IgY (H+L) Highly Cross Adsorbed Secondary Antibody, Alexa Fluor 647 (A78952; Invitrogen), Donkey Anti‐Goat IgG H&L (Alexa Fluor 488) (ab150129; Abcam), Donkey anti‐Rabbit IgG (H+L) Highly Cross‐Adsorbed Secondary Antibody, Alexa Fluor 488 (A‐21206; Invitrogen).

### Sample Processing for Single Cell Sequencing

Tongues were collected from 20 BL/6 mice, as previously described (in revision). CvP and FoP were collected using tweezers under a stereomicroscope. Tissues were minced and treated with TrypLE containing 10 µm Y‐27538 for up to 30 min. During the enzymatic digestion process, every 5 min, tissues were pipetted several times using a 1000 uL pipette tip. Single cells and small tissue clumps were collected in cold medium, and fresh TrypLE + 10 um Y‐27538 was added to further digest the remaining tissues. AT tissue was processed based on a previously described protocol.^[^ ^73^ ^]^ In brief, 300 – 400 µL of 1 mg mL^−1^ collagenase/dispase solution was injected under the lingual epithelium using a 27G needle, and tongues were incubated at 37 °C for 20 to 25 min. Then, the epithelium was peeled off and digested with TrypLE containing 10 µm Y‐27538 in the same manner as CvP and FoP tissues. ATEM and ATDM were collected and processed in a similar manner to the tissues. Single cells were washed twice with cold FACS buffer containing 1% FBS, 0.1 mm EDTA, and PBS. Cell pellets were resuspended in 500 µL cold FACS buffer, filtered through a 70 µm cell strainer, and then through a 40 µm FACS tube. Cells were stained with 5 µm Draq5 (424 101; Biolegend) and 1 µm 4′,6‐diamidino‐2‐phenylindole (DAPI, D9542; Sigma‐Aldrich) according to the manufacturer's protocols. Viable single cells (Draq5+/DAPI‐) were collected in PBS containing 1% BSA and 10 µm Y‐27538 using the SH800S Cell Sorter (Sony Biotechnology, CA, USA). Cell numbers were counted using a hemocytometer before and after cells were washed with PBS containing 1% BSA and 10 µm Y‐27538.

### Single Cell RNA Seq Analysis

Libraries from the sorted live single cells were prepared using a Chromium Next GEM Automated Single Cell 3′ Library and Gel Bead Kit v3 (10X Genomics) or GEM‐X Universal 3′ Gene Expression v4 4‐plex (10X Genomics)(“organoid validation dataset”), according to the manufacturer's instructions. Then, the libraries were sequenced using the NovaSeq6000 1092 (Illumina) flowcell. Sequencing reads were mapped to the mm10 genome, which is available from 10× genomics. Counts were generated using the Cell Ranger pipeline (v6.1.1 for tissue samples and v7.1.0 for organoid samples). Cell Ranger outputs were loaded onto R (v. 4.4.0) using the Seurat R package (v5.2.1).^[^ ^74^ ^]^ Based on distribution of “nCount_RNA” and sequencing depth, the following cutoff values for total number of counts per cell (“nCount_RNA”) were applied to each library: AT tissue (<1500, >25000), FoP (<2500, >20 000), CvP (<1500, >12 000), ATEM line 1 from “organoid discovery dataset” (<8000, >90 000), ATEM line 2 from “organoid validation dataset” (<9000, >75 000), PTEM line 1 from “organoid discovery dataset” (<7000, >50 000), PTEM line 2 from “organoid discovery dataset” (<5000, >40 000), PTEM line 3 from “organoid discovery dataset” (<6000, >45 000), and ATDM (<500, >65 000). Libraries of “FoP” and “CvP” samples were released in GSE274015.^[^ ^12^ ^]^ Single cells were excluded if they contained >20% of mitochondrial transcripts.

Counts were merged separately for the following three datasets: i) AT and PT tissue (CvP and FoP) samples, ii) ATEM line 1 and PTEM line 1 from the “organoid discovery dataset”, and iii) ATEM line 2 and PTEM line 2 and 3 from “organoid validation dataset”. Counts were log‐normalized with a factor of 10 000, scaled, and centered before the FindVariableFeatures function was used. S cell cycle, G2M cell cycle, and stress‐related genes were regressed out from the variable features. The following number of first PCA dimensions were used to generate UMAP clusters: 30 (AT and PT tissue), 5 (“organoid discovery dataset”), 20 (“organoid validation dataset”), and 5 (ATDM). A resolution of 0.4 (for AT and PT tissue; “organoid discovery dataset”), 0.2 (“organoid validation dataset”), and 0.1 (ATDM) was used to define clusters. Cells were annotated using known markers and cell cycle scores. In the AT and PT tissue dataset, T cells, dendritic cells, fibroblasts, monocytes, and endothelial cells were removed from downstream analysis except for CellChat analysis.

ScRNA‐seq datasets from different batches (ATEM and ATDM) or studies (AT tissue and Tabula Muris Tongue) were integrated using the RPCA method. S cell cycle, G2M cell cycle, and stress‐related genes were regressed out from the variable features. For the ATEM and ATDM integrated dataset, the first 5 dimensions were used to generate UMAP clusters. A resolution of 0.3 was used to define clusters. For the AT tissue and Tabula Muris Tongue integrated data (“tissue validation dataset”), pre‐processed Tabula Muris Tongue data were first obtained from “https://figshare.com/projects/Tabula_Muris_Transcriptomic_characterization_of_20_organs_and_tissues_from_Mus_musculus_at_single_cell_resolution/27733”.^[^ ^26^ ^]^ Then, counts of ‘Tongue‐10X_P7_10′, ‘Tongue‐10X_P4_1′, ‘Tongue‐10X_P4_0′ microfluidic emulsion were merged. Next, counts of AT tissue and Tabula Muris Tongue were integrated using 19 770 genes that are shared between the datasets, and the first 20 dimensions were used to generate UMAP clusters. A resolution of 0.2 was used to define clusters, and clusters were annotated based on ‘free_annotation’ metadata from “annotations_droplet.csv”. Langerhans cells were removed from the downstream analysis.

DEG analysis was performed using the FindAllMarkers function with the following criteria: log fold change value of ≥ 1.5 and adjusted p value of ≤ 0.05. AT and PT gene signatures were obtained by using FindMarkers function on two groups that are region‐restricted (“4 Cycling basal cells4”,“2 Cycling basal cells2”, “6 Slow cycling basal cells2”, “7 Slow cycling basal cells3”, “11 Keratinocytes1”, “8 Lgr6+/Lgr5‐ cells” versus “3 Cycling basal cells3”, “5 Slow cycling basal cells1”, “12 Keratinocytes2”, “13 Keratinocytes3”, “9 Lgr6+/Lgr5+ cells”). Then, the DEGs meeting the following criteria were selected as AT gene signature and PT gene signature: log fold change value of ≥ 1.5, adjusted p value of ≤ 0.05, and >3 fold‐difference in percentage of cells expressing the gene between the two groups (Tables S2 and S3, Supporting Information). 71 DEGs meeting the following criteria were selected as *Krt23+/Sbsn+* cluster gene signature: log fold change value of ≥ 1.5, adjusted p value of ≤ 0.05, and >3 fold‐difference in percentage of cells expressing the gene between the two groups. Violin plots, feature plots, and dot plots were generated using scCustomize (v.3.0.1).

Mouse gastrulation and early embryogenesis scRNA‐seq data were retrieved as a SingleCellExperiment object from the MouseGastrulationData R package (v.1.18.0).^[^ ^25^ ^]^ Dot plots were visualized using the dittoSeq R package (v.1.16.0).^[^ ^75^ ^]^

Scores for gene signatures were calculated using the AddModuleScore Seurat function.

### SCENIC Analysis

Gene regulatory network analysis was performed using a command‐line version of pySCENIC.^[^ ^29^ ^]^ In brief, gene co‐expression networks were identified using GRNBoost2, and regulons were defined using the ctx function in 3 datasets: i) AT, PT tissue, ATEM, and PTEM organoids, ii) ATEM and ATDM organoids, and iii) AT tissue and Tabula Muris tongue dataset. Regulon activity per cell was calculated using the aucell function and merged with a relevant Seurat object. Network visualization of TFs and their regulons was performed using Cytoscape (v.3.10.2).^[^ ^76^ ^]^ TFs that are predicted to regulate >5% of genes in the AT or PT gene signatures were defined as AT TFs or PT TFs, respectively. RSS was evaluated for each cluster or cell type, as previously described.^[^ ^30^ ^]^ To evaluate cell type‐specific RSS, a new metadata file was compiled for cycling basal cells (‘1 Cycling basal cells1’, ‘2 Cycling basal cells2’, ‘3 Cycling basal cells3’, ‘4 Cycling basal cells4’), basal cells (‘5 Basal cells1’, ‘6 Basal cells2’, ‘7 Basal cells3’), and keratinocytes (“11 Keratinocytes1”, ’12 Keratinocytes2’, ’13 Keratinocytes3’). A Venn diagram was created using the VennDiagram R package (v.1.7.3). Regulon activity matrices were visualized using the ComplexHeatmap R package (v.2.20.0).

### CellChat Analysis

Cell‐cell communication networks in AT tissue or ATEM were inferred using the CellChat R package (v2.1.2).^[^ ^36^ ^]^ “All signaling” and “Secreted signaling” from CellChatDB were used.

### Gene Set Enrichment Analysis

GSEA was performed using clusterProfiler (v4.12.6)^[^ ^77^ ^]^ and the Gene Ontology Biological Process (GOBP) resource. AT TF and PT TF regulons, differentially expressed genes for cycling basal cells, basal cells, and keratinocytes were used as inputs.

### Statistical Analysis

GraphPad Prism version 10 or R was used for statistical analyses. The Mann‐Whitney U test was used to assess the statistical significance of differential regulon activity between ATEM and ATDM. Two‐tailed Student *t*‐tests were performed to compare qPCR results between ATEM and PTEM, and between ATEM and ATDM. Bar graph data are presented as the mean ± SEM unless indicated otherwise.

### Ethics Approval Statement

All animal experiments were performed under the guidelines and approval of the animal welfare committee of the Netherlands Cancer Institute and Hubrecht Institute (KNAW).

## Conflict of Interest

J.S. is an employee and shareholder of Givaudan International SA. C.W. is an employee of Givaudan International SA. H.C. and M.v.d.W are inventors on patents held by the Royal Netherlands Academy of Arts and Sciences that cover organoid technology. H.C. is now head of pharma Research and Early Development (pRED) at Roche, Basel, Switzerland. H.C.’s full disclosure is given at https://uu.nl/staff/JCClevers/. All other authors declare that they have no competing interests.

## Supporting information



Supporting Information

## Data Availability

The raw and processed single cell RNA sequencing data are available in Gene Expression Omnibus (accession number: GSE307623). The codes used in this study are available in https://urldefense.com/v3/__https://github.com/PMC-Clevers/Mouse_anterior_tongue__;!!N11eV2iwtfs!v5qH3VrpmfHng1v8nakn4wHYYMDqVyiyNlRoivQG7J4hvF6MN9Sb_6H2lEvDob0pYlAltf3pp7EFApFLp0HW$.
