# Supplementary material for: Organoid Modeling of Mouse Anterior Tongue Epithelium Reveals Regional and Cellular Identities
Source: Adv Sci (Weinh). 2025 Sep 29;12(46):e06738. doi: 10.1002/advs.202506738 (PMC12697779; doi:10.1002/advs.202506738)
Supplement: Supplementary file 1 — Supporting Information [file ADVS-12-e06738-s001.docx]

Supporting Information

**Organoid Modeling of Mouse Anterior Tongue Epithelium Reveals Regional and Cellular Identities**

*Seok-Young Kim*, Laurens Verweij*, Lin Lin, Johan H. van Es, Jay Slack, Chris Winkel, Tito Candelli, Philip Lijnzaad, Thanasis Margaritis, Gerben E. Breimer, Karin Sanders, Marc van de Wetering**, Hans Clevers***


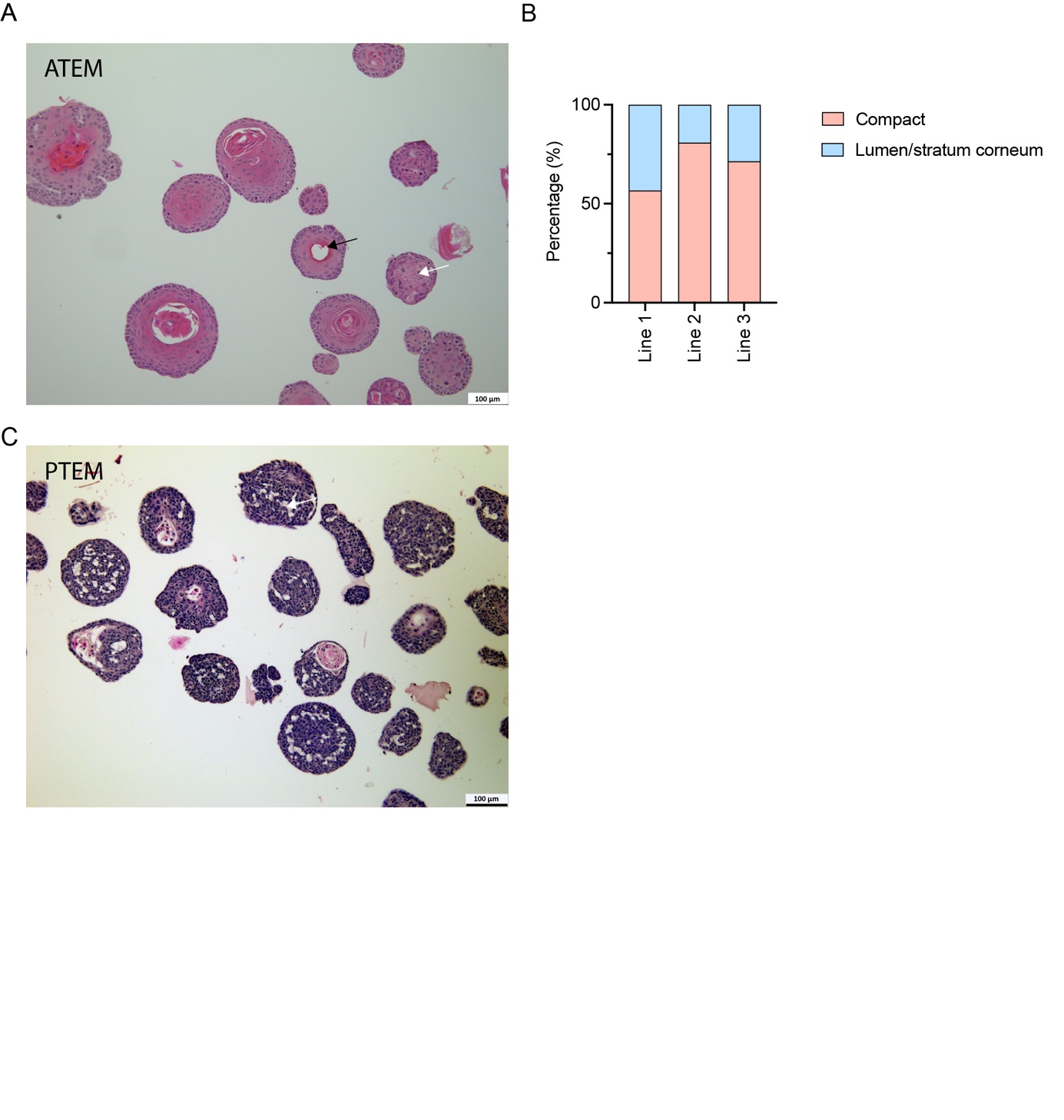


Figure S1. Histological analysis of ATEM and PTEM. A) Representative hematoxylin and eosin image of ATEM. The black arrow indicates an individual organoid harboring a lumen. Stratum corneum and lumen in ATEM frequently stained red with eosin. The white arrow indicates an individual organoid with compact morphology. B) Distribution of organoid morphologies across biological triplicates of ATEM. C) Representative hematoxylin and eosin image of PTEM. The white arrow indicates an individual organoid harboring a (pseudo)gland-like structure. A,C) Results from biological triplicates are shown. Scale bar, 500 μm.


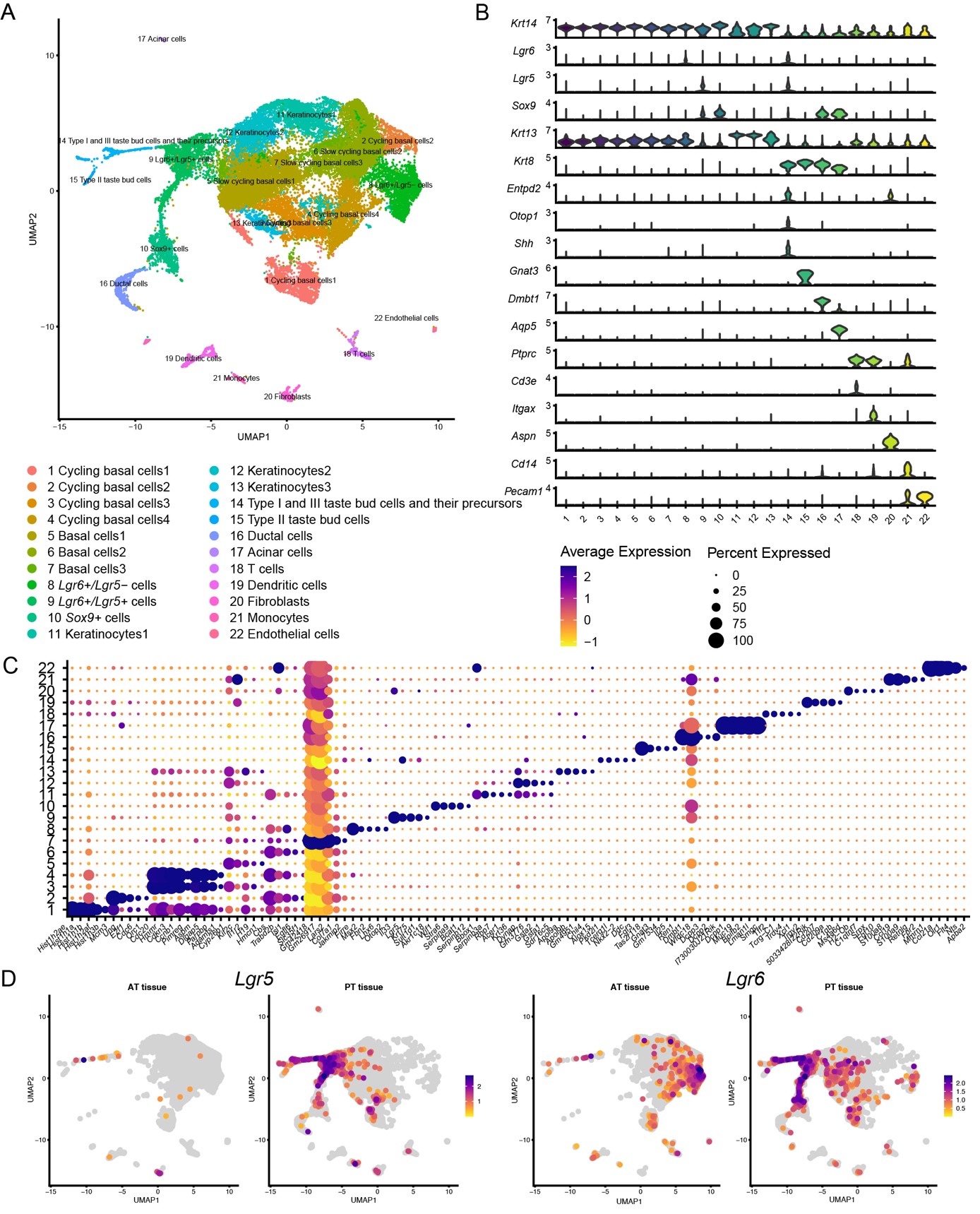


Figure S2. Single-cell characterization of mouse anterior and posterior dorsal tongue tissues. A) UMAP visualization of 22 cell types in the tissue single-cell RNA sequencing data. B) A violin plot showing expression of known markers for each cluster in this dataset. C) A dot plot showing cluster specific markers in this dataset. Top 5 markers are shown. D) UMAP showing *Lgr5* and *Lgr6* expression in AT and PT tissues. AT = anterior tongue, PT = posterior tongue.


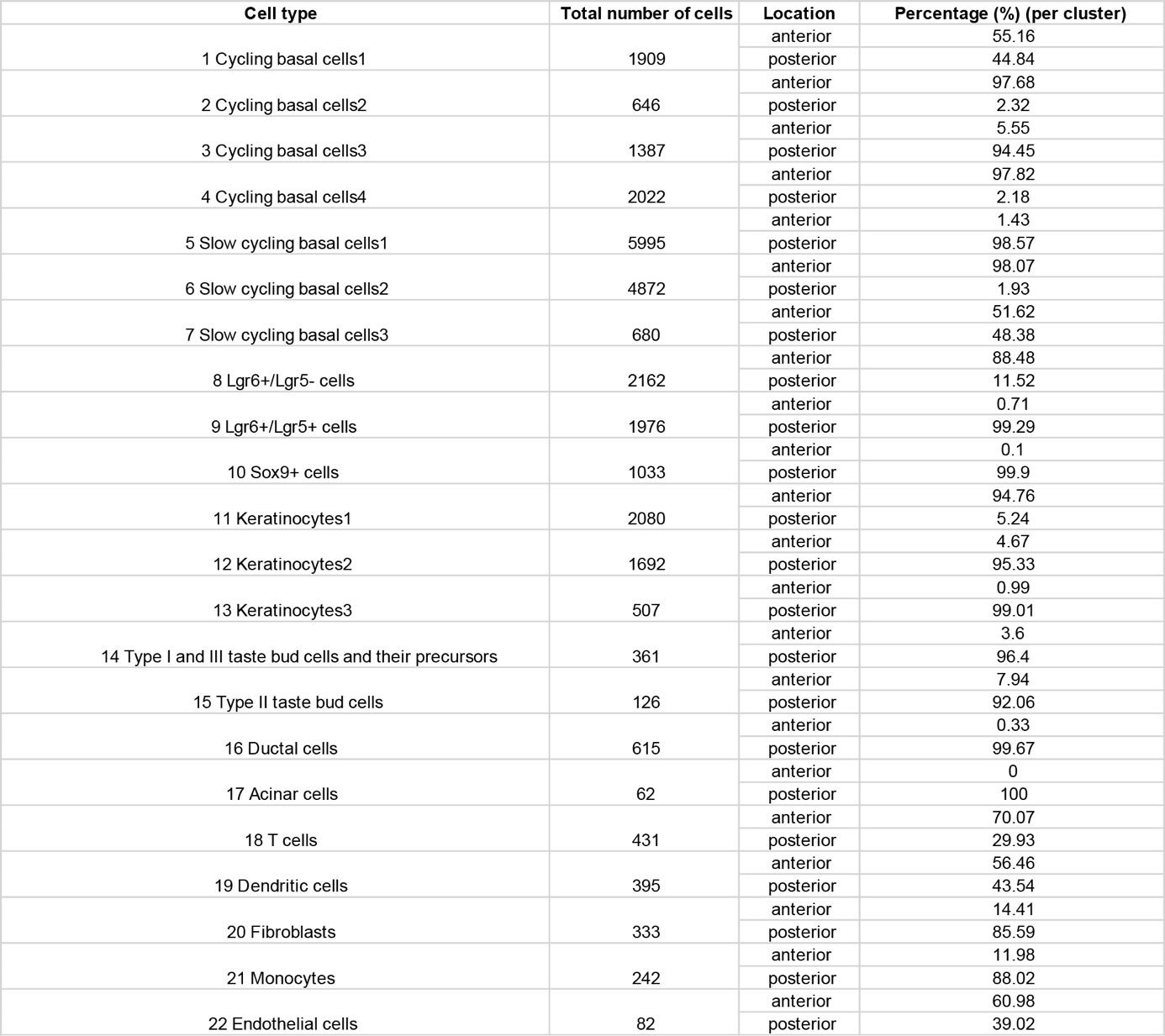


Figure S3. Regional composition of UMAP clusters in the AT and PT tissue scRNA-seq data.


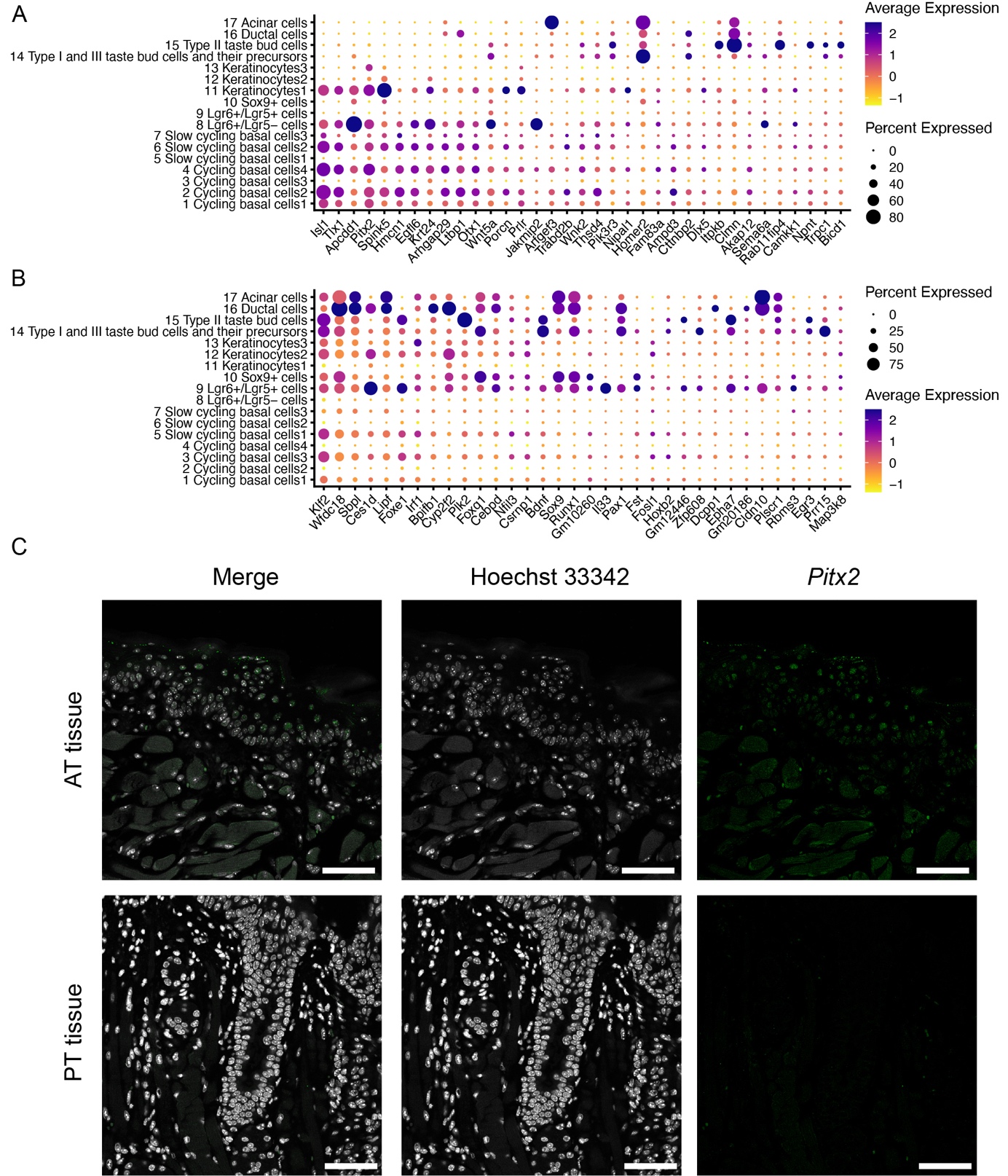


Figure S4. Expression of region-specific genes in AT and PT tissue. A) A dot plot based on the tissue scRNA-seq data showing expression patterns of genes included in AT gene signature. 35 genes that have 4-fold difference in the percentage of cells expressing the gene between AT and PT tissues are shown. B) A dot plot showing expression patterns of genes included in PT gene signature. 34 genes that have 4-fold difference in the percentage of cells expressing the gene between AT and PT tissues are shown. AT = anterior tongue; PT = posterior tongue. C) Representative fluorescent immunohistochemistry images showing expression of Pitx2 in AT and PT tissues. Hoechst 33342 marks the nuclei. Results from biological duplicates are shown. Scale bar, 50 μm.


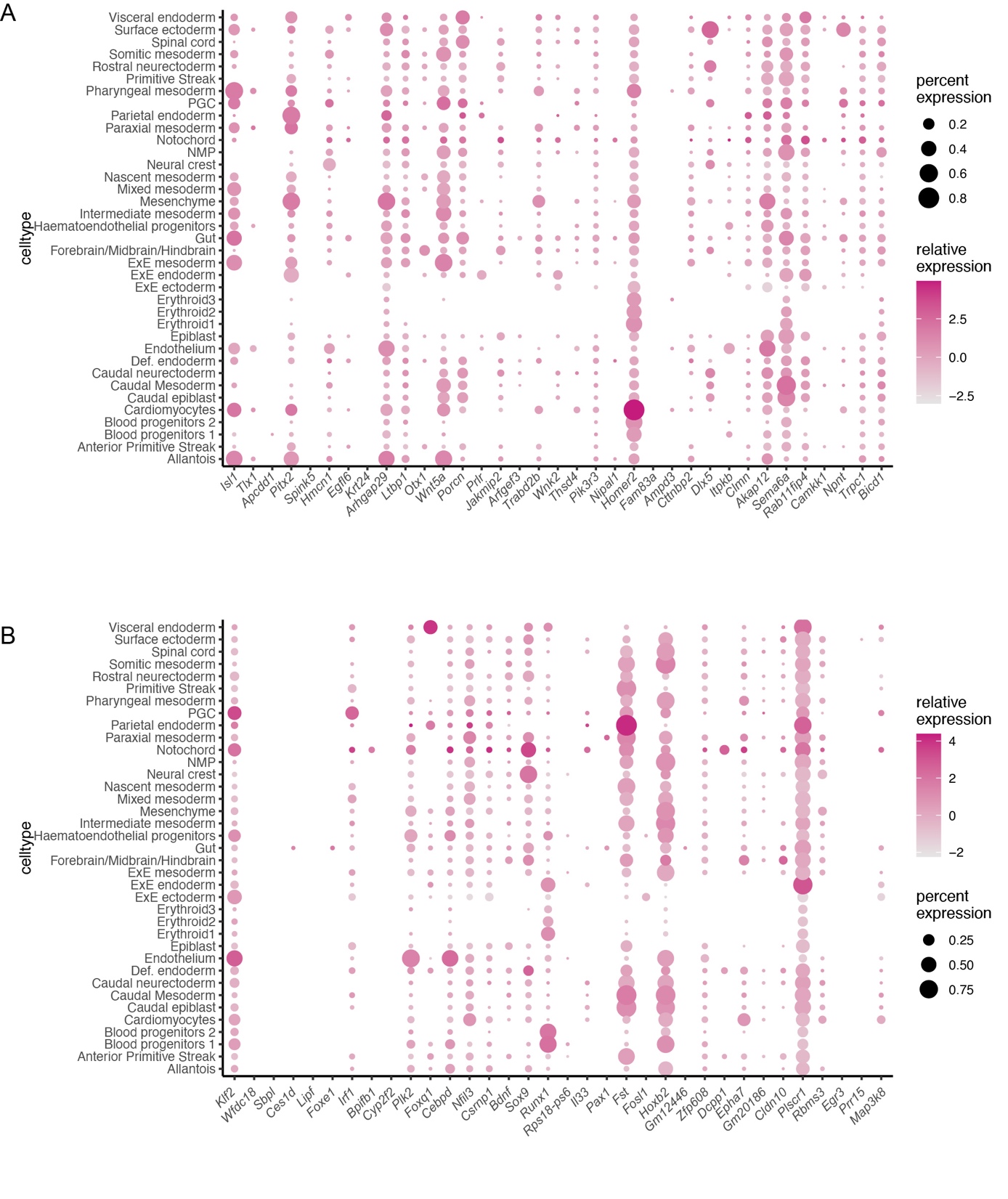


Figure S5. Expression patterns of AT and PT specific genes during mouse embryogenesis (E6.5 – E8. A) A dot plot showing AT-specific genes. B) A dot plot showing PT-specific genes. AT = anterior tongue; PT = posterior tongue.


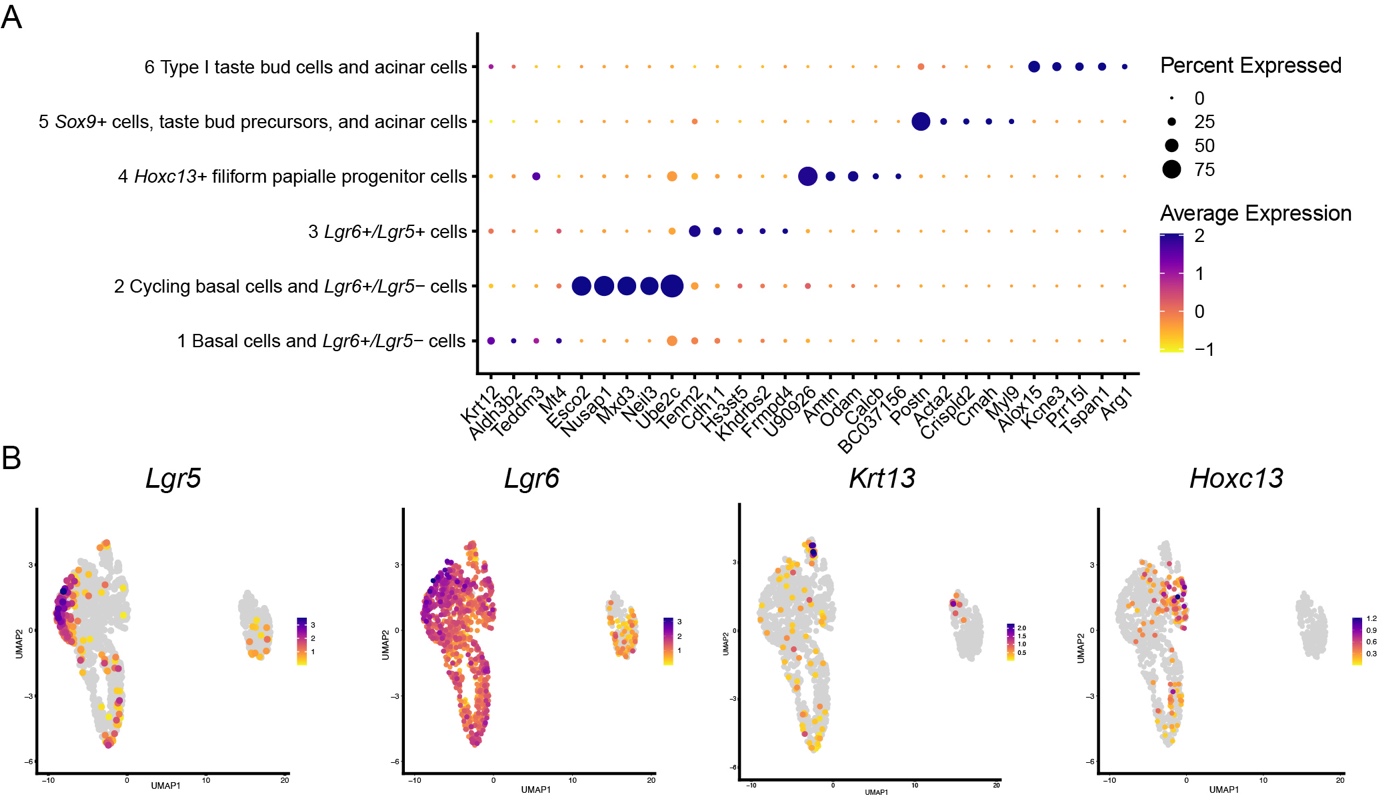
Figure S6. Single-cell characterization of ATEM and PTEM. A) A dot plot showing cluster-specific markers in ATEM and PTEM single-cell RNA sequencing data. Top 5 markers for each cluster are shown. B) UMAP plots showing expression patterns of *Lgr5, Lgr6, Krt13,* and *Hoxc13* in this dataset. ATEM = anterior tongue organoids cultured in expansion medium; PTEM = posterior tongue organoids cultured in expansion medium.


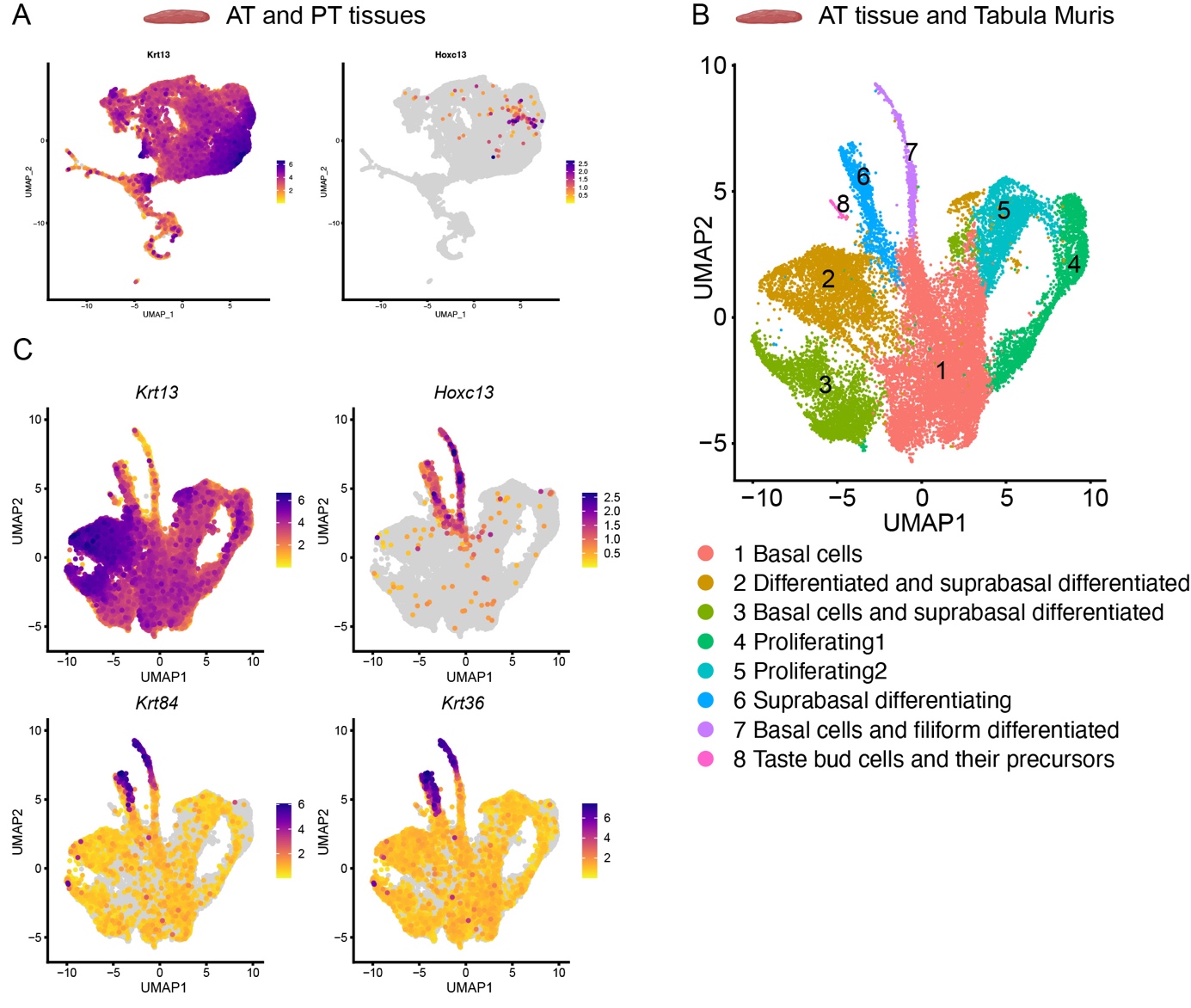


Figure S7. Integrated analysis of AT tissue and Tabula Muris Tongue single-cell RNA sequencing dataset. A) UMAP plots showing expression of *Krt13* and *Hoxc13* in the AT and PT tissue scRNA-seq dataset. B) UMAP visualization of 8 epithelial cell clusters in AT tissue and Tabula Muris Tongue dataset. Cells were annotated according to metadata of Tabula Muris data. Cell types are color-coded. C) UMAP plots showing expression patterns of *Krt13, Hoxc13, Krt84,* and *Krt36*. AT = anterior tongue, PT = posterior tongue.


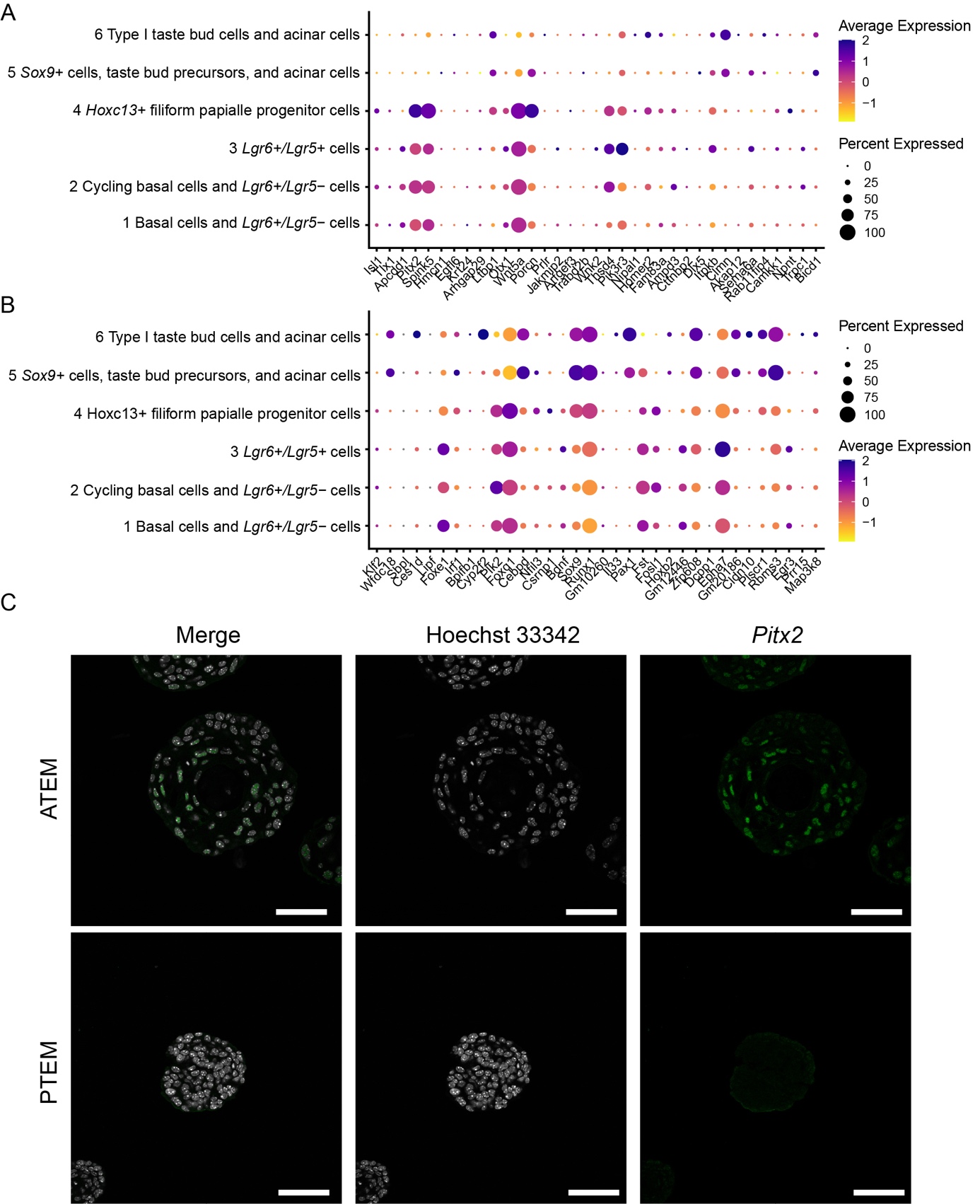


Figure S8. Expression of region-specific genes in ATEM and PTEM. A) A dot plot from the organoid discovery scRNA-seq data showing expression patterns of genes included in AT gene signature. 35 genes that have 4-fold difference in the percentage of cells expressing the gene between AT and PT tissues are shown. B) A dot plot showing expression patterns of genes included in PT gene signature. 34 genes that have 4-fold difference in the percentage of cells expressing the gene between AT and PT tissues are shown. C) Representative fluorescent immunohistochemistry images showing expression of Pitx2 in ATEM and PTEM. Hoechst 33342 marks the nuclei. Results from biological duplicates are shown. Scale bar, 50 μm. ATEM = anterior tongue organoids cultured in expansion medium; PTEM = posterior tongue organoids cultured in expansion medium.


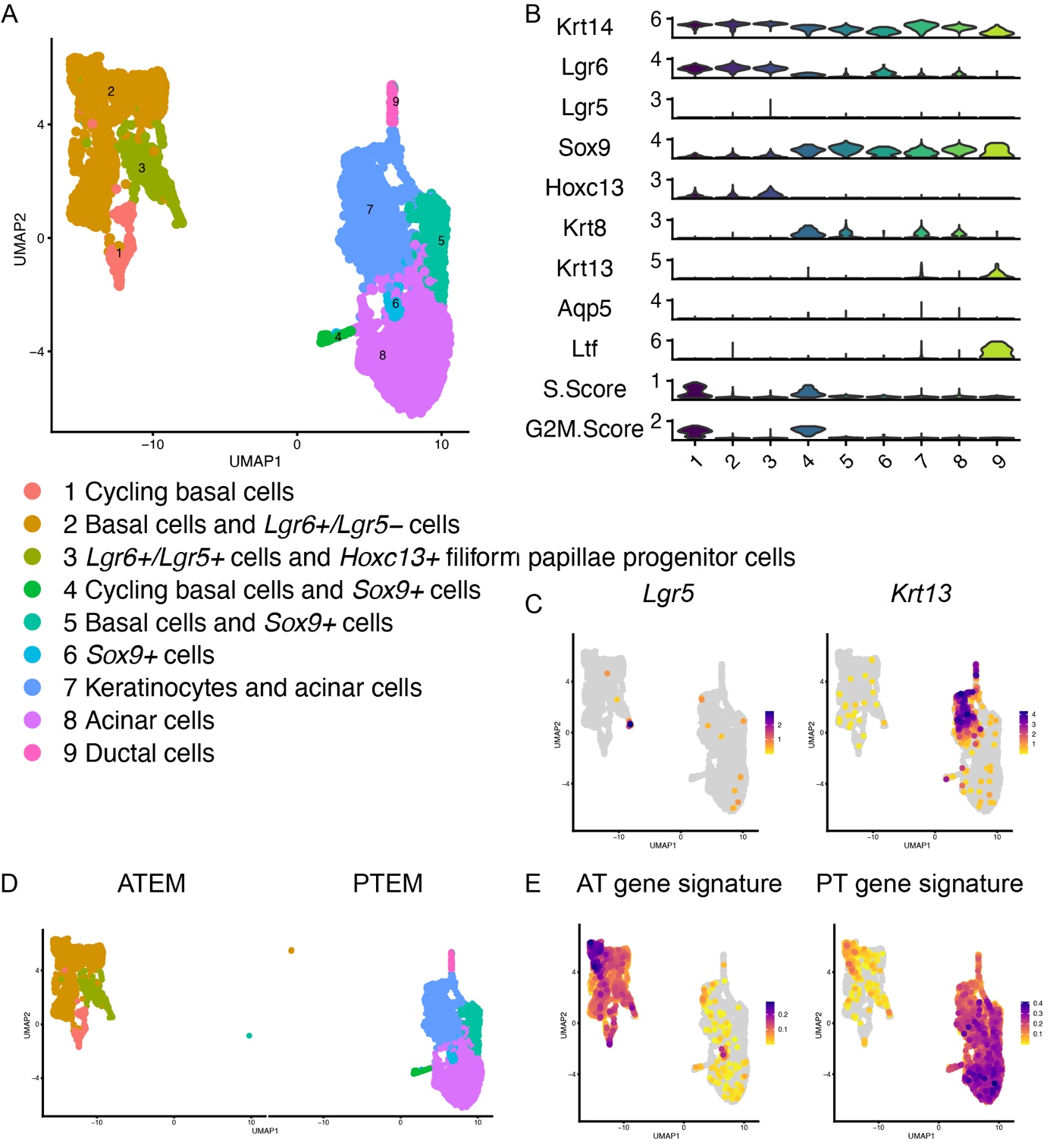


Figure S9. Validation dataset from scRNA-seq of ATEM and PTEM. A) UMAP visualization of 9 clusters in biological replicates of ATEM (n = 1) and PTEM (n = 2). B) A violin plot showing expression of known markers and cell cycle scores for each cluster C) UMAPs showing Lgr5 and Krt13 expression patterns. D)UMAPs split by tissue origins. E) UMAPs showing AT and PT gene signature patterns.


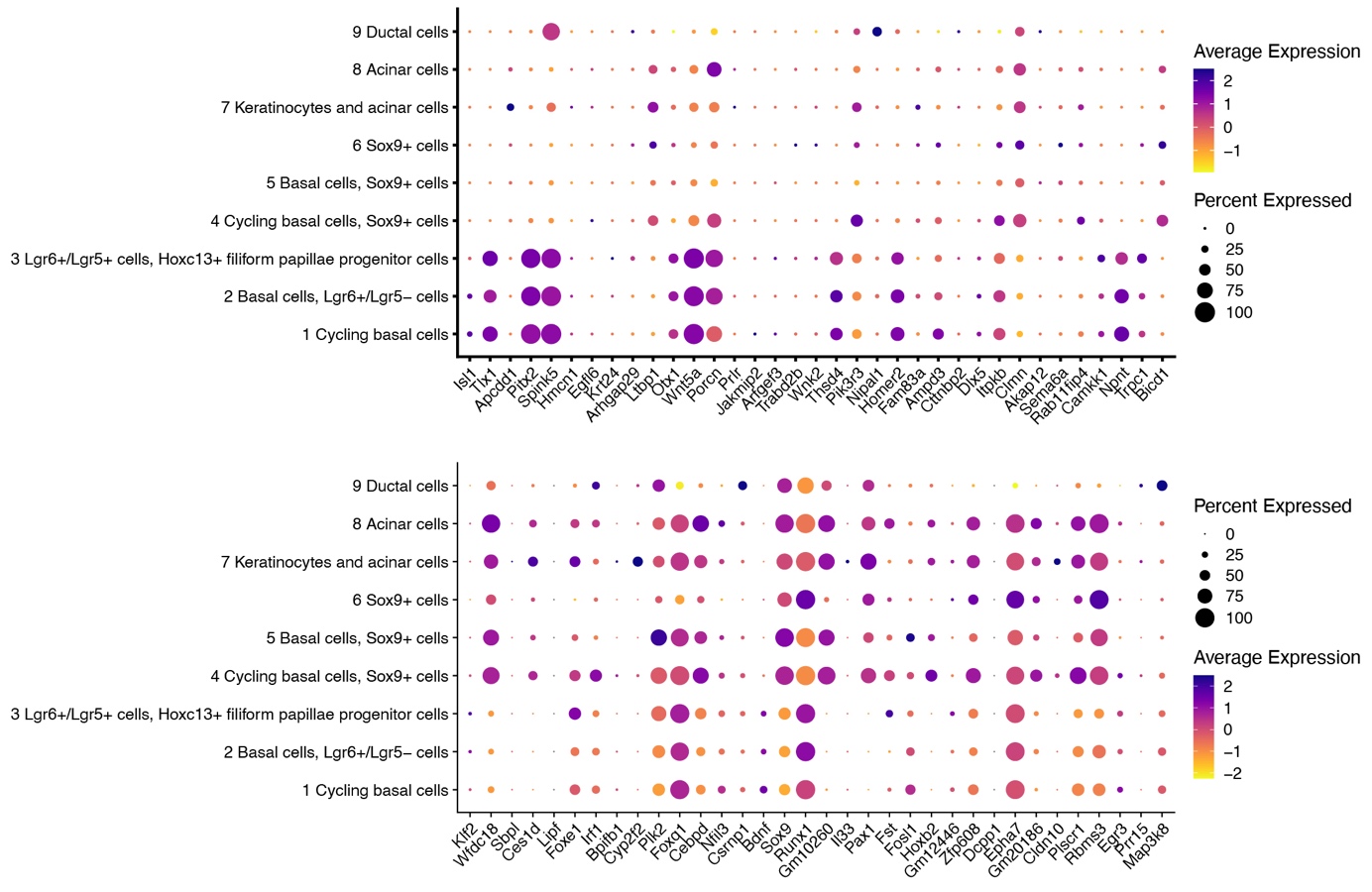


Figure S10. Expression of region-specific genes in biological replicates of ATEM and PTEM scRNA-seq data. A) A dot plot from the organoid validation scRNA-seq data showing expression patterns of genes included in AT gene signature. 35 genes with a 4-fold difference in the percentage of cells expressing the gene between AT and PT tissues are shown. B) A dot plot showing expression patterns of genes included in PT gene signature. 34 genes with a 4-fold difference in the percentage of cells expressing the gene between AT and PT tissues are shown. ATEM = anterior tongue organoids cultured in expansion medium; PTEM = posterior tongue organoids cultured in expansion medium.


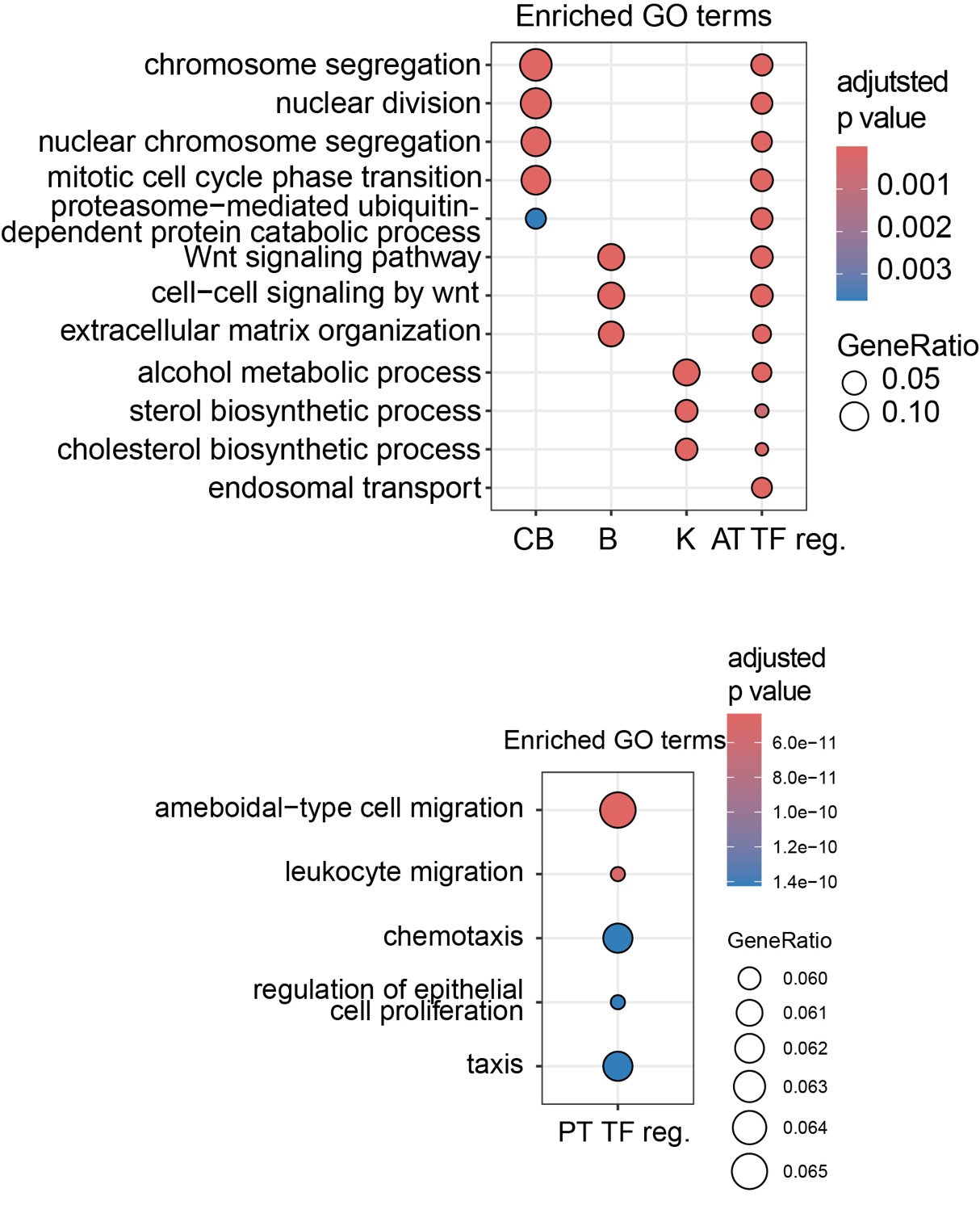


Figure S11. Gene set enrichment analysis showing biological processes that are associated with cycling basal cells, basal cells, keratinocytes, regulons of AT TFs (top panel) and or regulons of PT TFs (bottom panel).


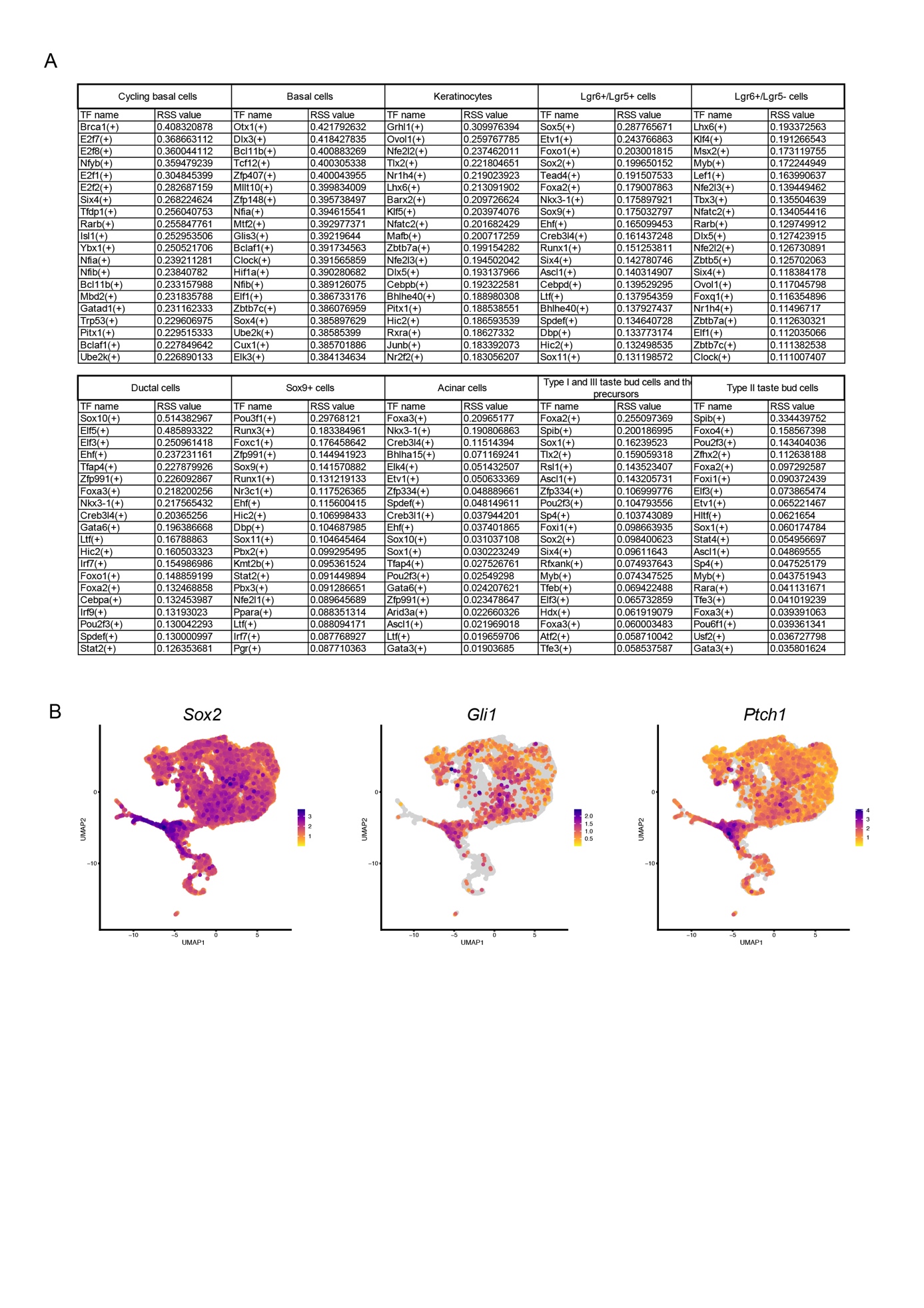


Figure S12. Cell type-specific regulons in AT and PT tissues. A) A table showing regulon-specific scores (RSS) in the described clusters. A high RSS value of a regulon indicates its specificity against the cluster. The top 20 TFs in each cluster are shown with RSS values per TF. To identify TFs which are specific to cycling basal cells, basal cells, and keratinocytes independent of their regional identity, we first re-assigned cluster labels in the UMAP analysis. For example, clusters labeled “1 Cycling basal cells”, “2 Cycling basal cells”, “3 Cycling basal cells”, “4 Cycling basal cells” (Figure 2A in the revised manuscript) were grouped under a single label “Cycling basal cells”. Then, RSS values were examined in the re-assigned clusters. B) UMAP plots showing expression of *Sox2*, *Gli1*, and *Ptch1*. AT = anterior tongue; PT = posterior tongue.


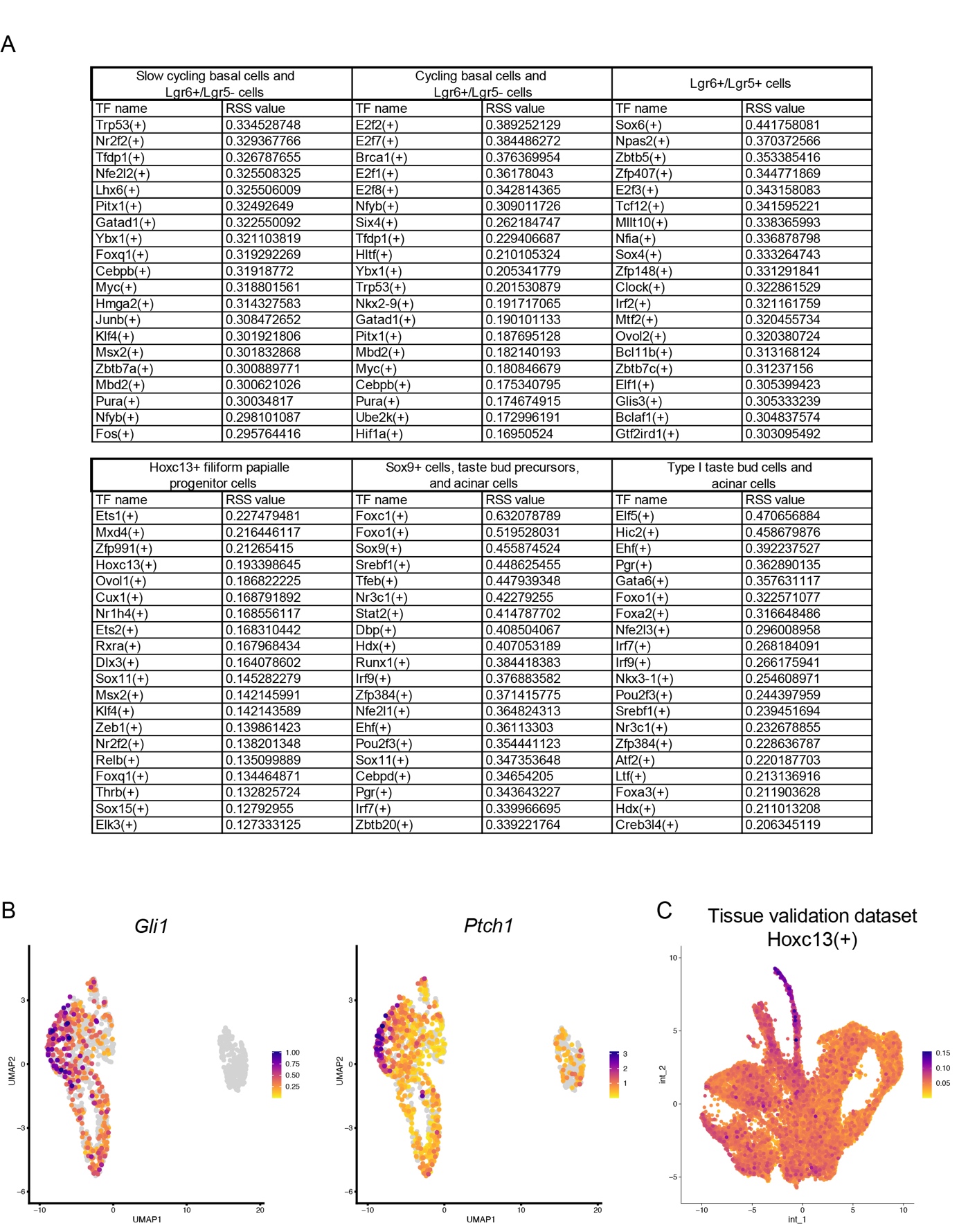


Figure S13. Cell type-specific regulons in ATEM and PTEM. A) A table showing regulon-specific scores (RSS) in the described clusters. The top 20 TFs in each cluster are shown with RSS values per TF. B) Gene expression of *Gli1* and *Ptch1*. C) Regulon activity of *Hoxc13* in the tissue validation dataset. ATEM = anterior tongue organoids cultured in expansion medium; PTEM = posterior tongue organoids cultured in expansion medium.


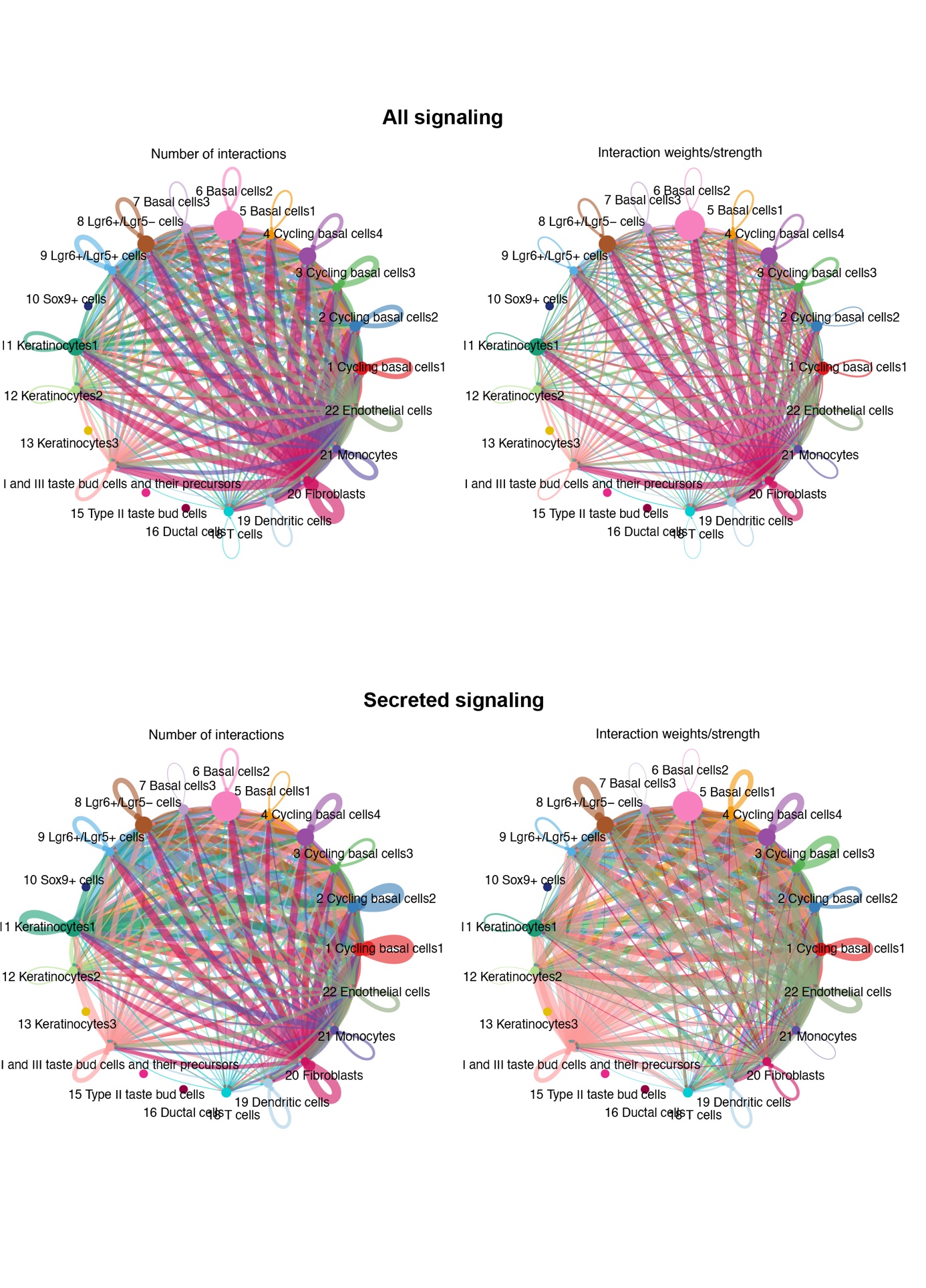


Figure S14. CellChat analysis reveals intercellular communications networks in AT tissue. Plots showing a number or weights/strength of predicted signalings in AT tissue. Two types of interactions are shown: 1) ‘All signaling’, including secreted signaling, ECM-receptor interactions, cell-cell interactions, and non-protein signaling; and 2) ‘Secreted signaling’, including only secreted signaling. AT = anterior tongue; ATEM = AT organoids cultured in expansion medium.


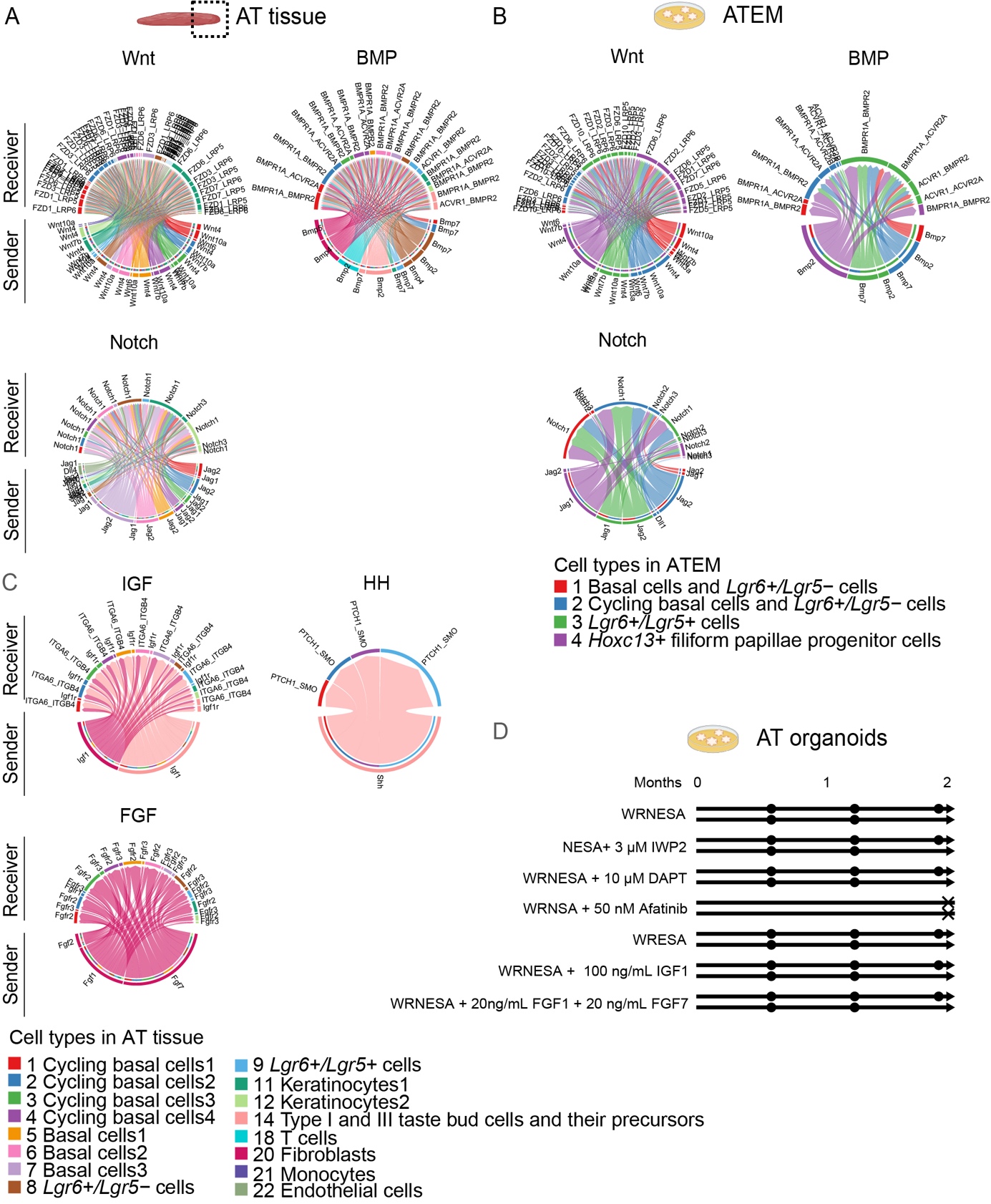


Figure S15. Mouse dorsal anterior tongue tissue and organoids largely share

intercellular communication networks. Cellchat analysis was performed on AT tissue

and ATEM scRNA-seq data. A) Chord diagrams showing senders and receivers of

the described signaling pathways in AT tissue. B) Chord diagrams showing senders

and receivers of the described signaling pathways in ATEM. Cell types are color-coded. C) Chord diagrams showing senders and receivers of the tissue-specific

signaling pathways in AT tissue. D) AT organoids were cultured in the indicated medium compositions upto 2 months. W, Wnt-surrogate. R, R-spondin 1. N, Noggin. E, EGF. S, SAG. A, A83-01. IWP2, a Wnt pathway inhibitor. DAPT, a Notch inhibitor. Afatinib, a pan-ERBB inhibitor. Each line represents a biological replicate. Circle indicates a passaging point. The arrow indicates cells were viable at the end of the experiment, whereas the cross indicates loss of viability.


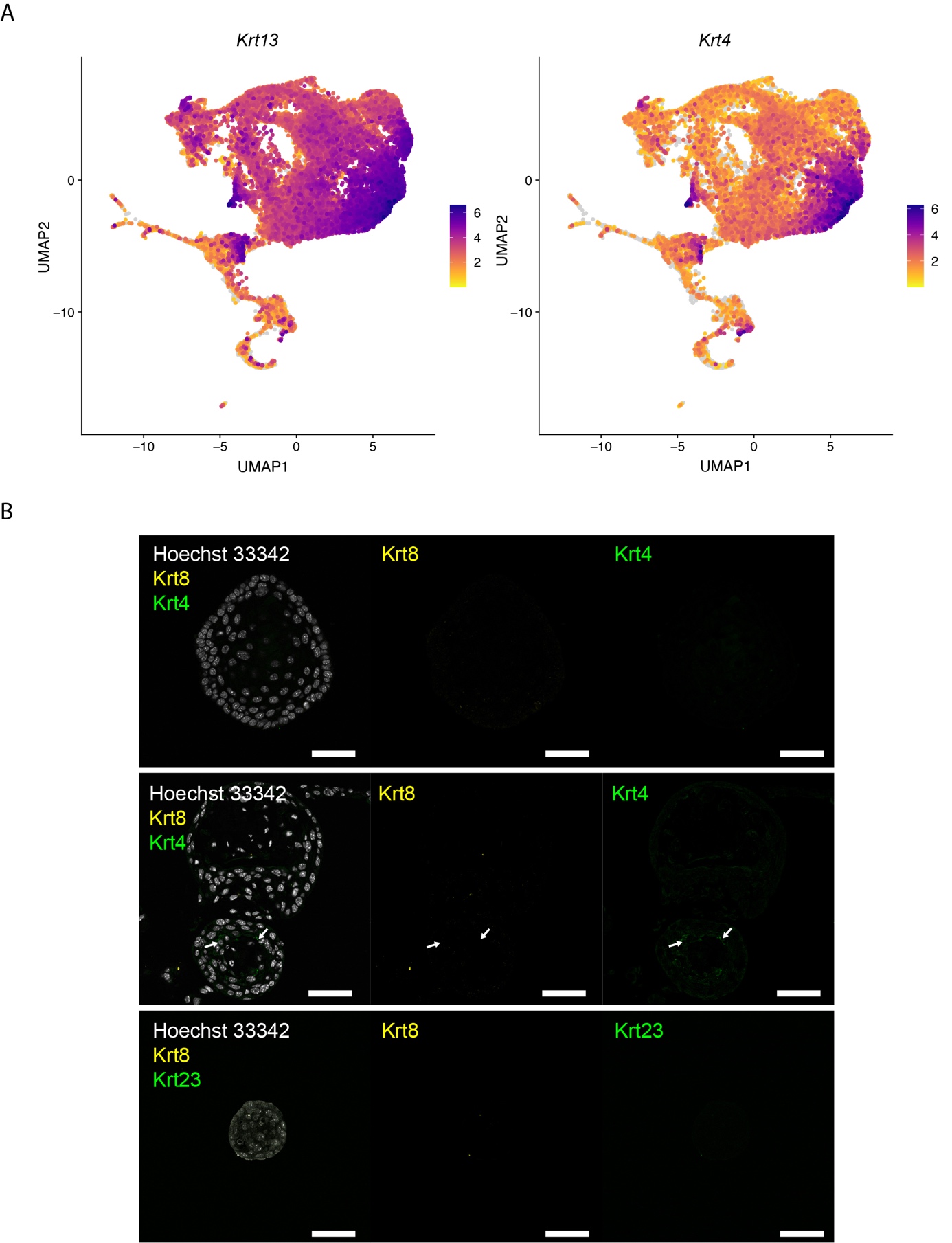


Figure S16. Expression of markers for keratinocytes in ATEM. A) UMAP showing expression of *Krt13* and *Krt4*, keratinocyte markers, in anterior and posterior tongue tissue single-cell RNA sequencing data. B) Representative fluorescent immunohistochemistry images showing expression of Krt8 (yellow), Krt4 (green, top and middel panel), and Krt23 (green, bottom panel) in ATEM. Some organoids with lumens displayed occasional and weak Krt4 staining (white arrows). Results from biological duplicates are shown. Hoechst 33342 (white) marks the nuclei. Scale bar, 50 μm.


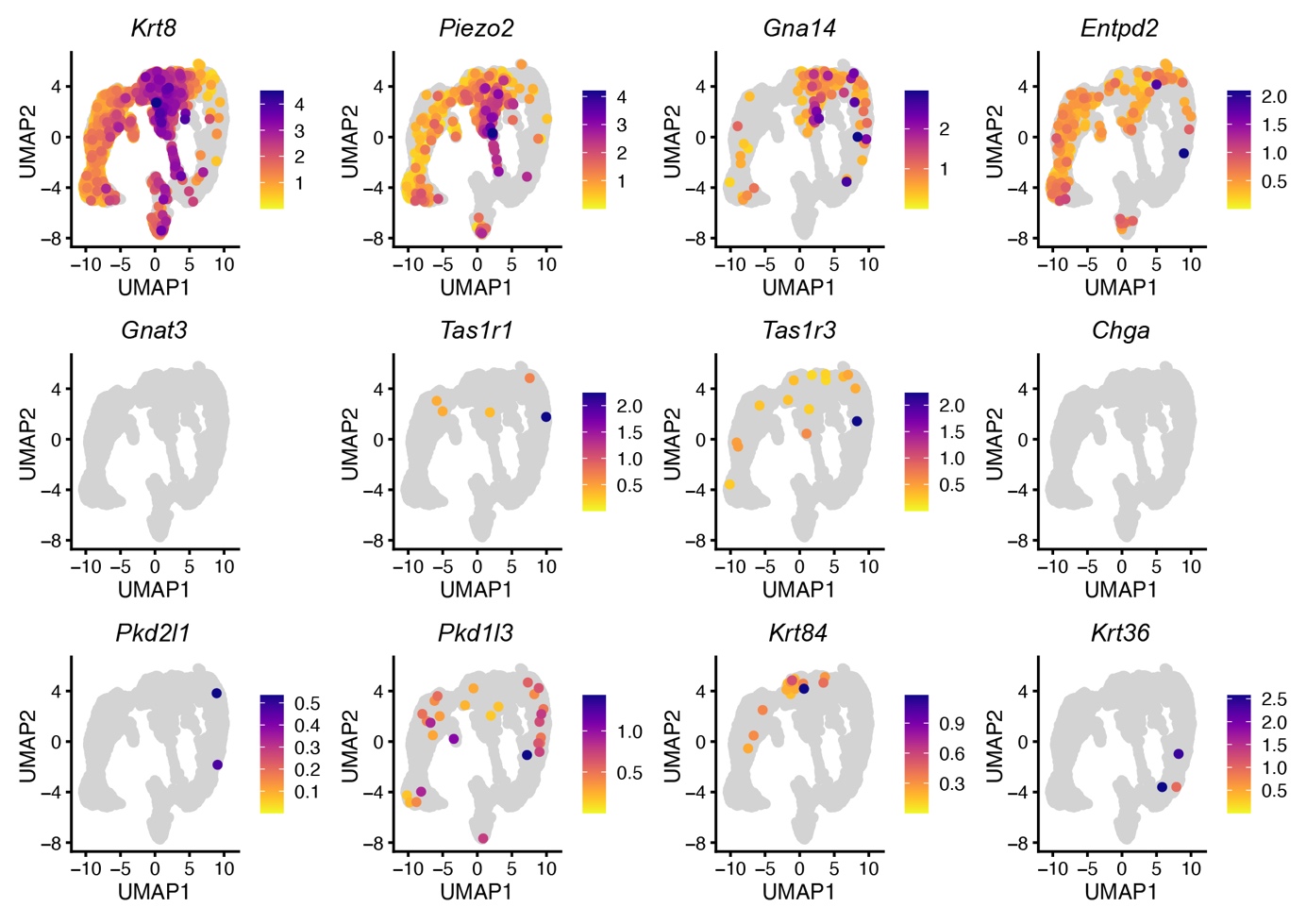


Figure S17. Expression of taste bud cell, Merkel-like cell, and mature filiform papillae cell markers in ATDM.


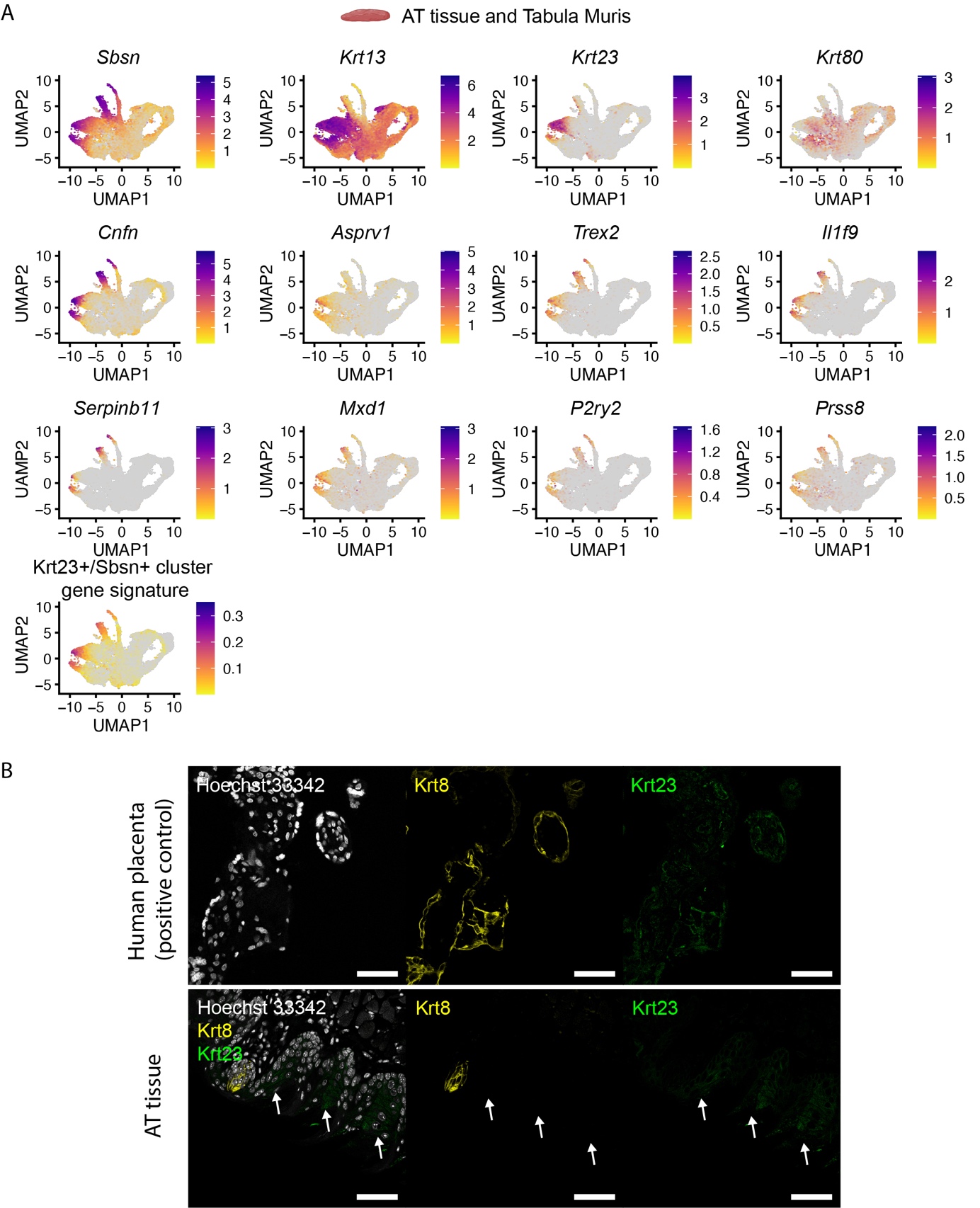


Figure S18. *Krt23+*/*Sbsn*+ keratinocytes are present in the AT tissue. A) Expression of markers and gene signature score in the validation dataset. B) Representative fluorescent immunohistochemistry images showing expression of Krt8 (yellow) and Krt23 (green) in ATEM. Hoechst33342 (white) marks the nuclei. Human placenta was used as a positive control. Scale bar, 50 μm.AT = anterior tongue.


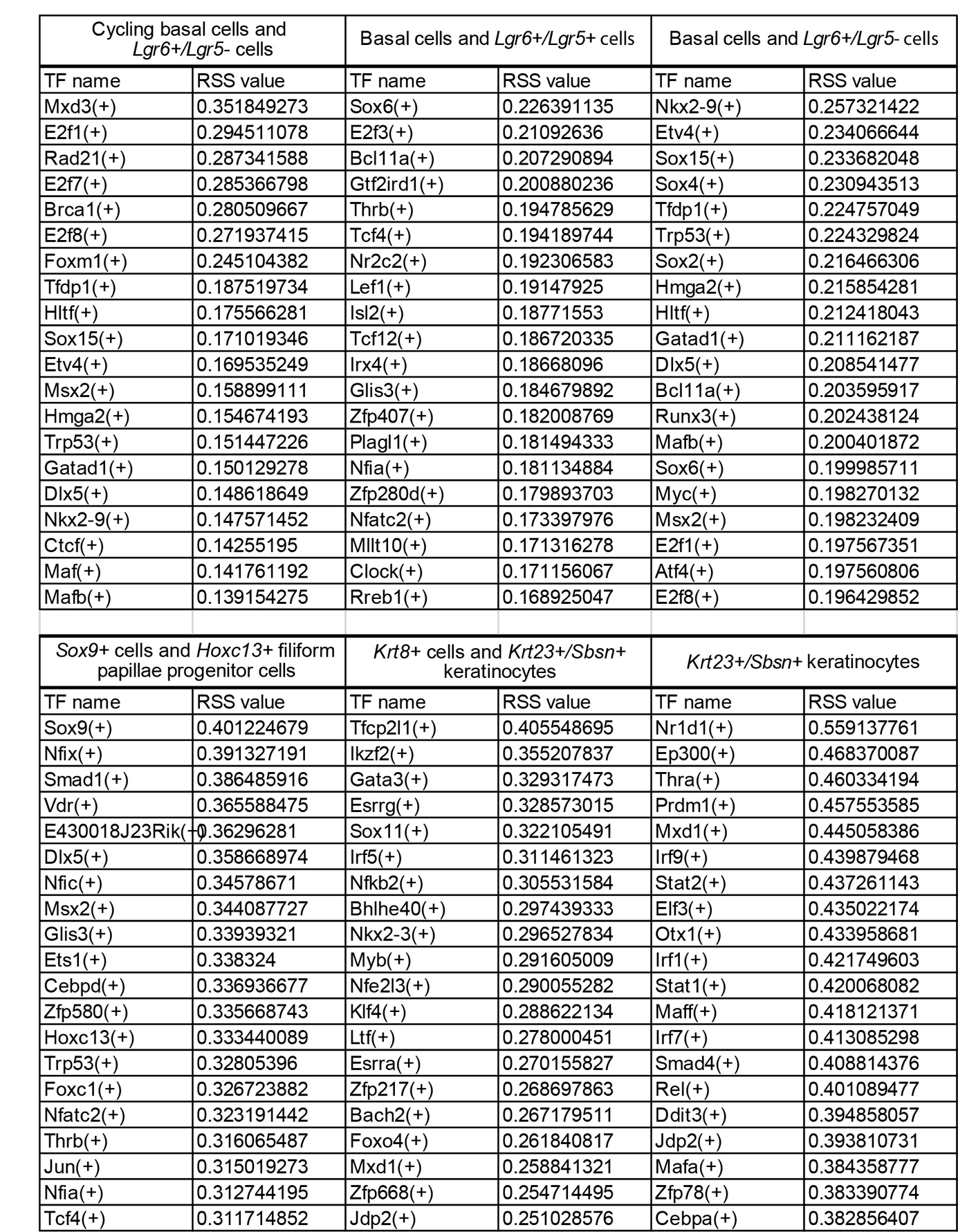


Figure S19. Cluster-specific regulons in the ATEM and ATDM integrated dataset. The top 20 TFs in each cluster are shown with regulon-specific scores (RSS) values per TF.


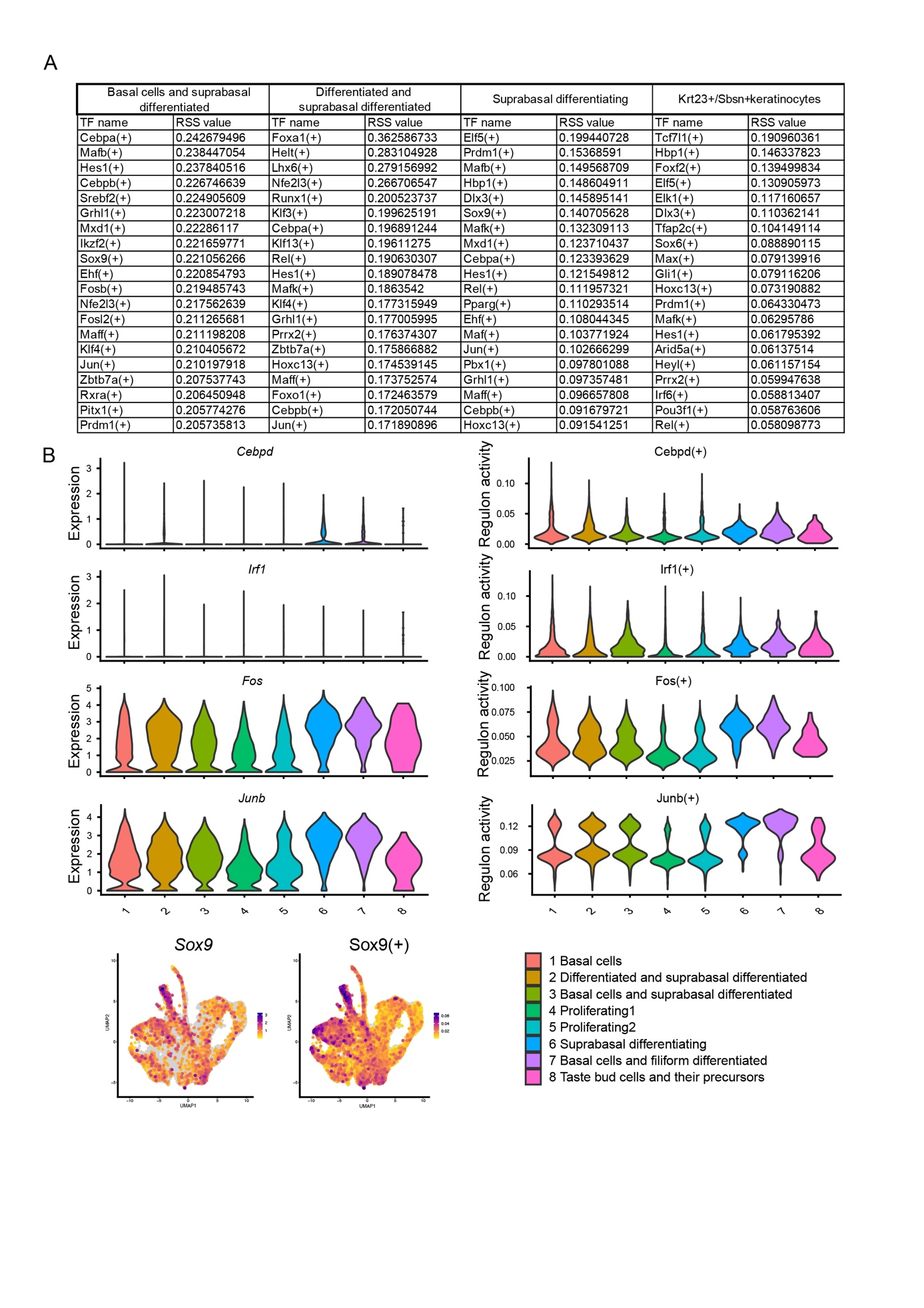


Figure S20. Gene regulatory networks that are associated with epithelial cell differentiation in the validation dataset. A) A table showing regulon-specific scores (RSS) in the described clusters. The top 20 TFs in each cluster are shown with RSS values per TF. B) Violin plots and UMAP plots showing expression and activity of the described transcription factors (TFs). Clusters are color-coded.
